# Supplementary material for: Insights into the Phylogeny and Enigmatic Mitochondrial Biology of Eustigmatophyte Algae From Over 50 Newly Sequenced Organellar Genomes
Source: Genome Biol Evol. 2026 Jul 7;18(8):evag169. doi: 10.1093/gbe/evag169 (PMC13449250; doi:10.1093/gbe/evag169)
Supplement: Supplementary file 1 [file evag169_supplementary_data.zip › Supplementary_Dataset_S2.pdf]

Insights into the phylogeny and enigmatic mitochondrial biology of eustigmatophyte algae from over 50 newly sequenced organellar genomes

Michal Richtář[1], Eliška Klapuchová[1], Tatiana Yurchenko[1], Karen P. Fawley[2], Marvin W. Fawley[2], Dovilė Barcytė[1], Karin Jaške[1], Benjamin M. Wolf[3], Fay-Wei Li[4,5], Tereza Ševčíková[1], Marek Eliáš[1]*

[1]Department of Biology and Ecology, Faculty of Science, University of Ostrava, 70900 Ostrava, Czech Republic
[2]Division of Natural Sciences and Mathematics, University of the Ozarks, Clarksville, Arkansas, 72830, USA
[3]Department of Biology, Washington University in St. Louis, 1 Brookings Dr., St. Louis, MO, USA
[4]Plant Biology Section, Cornell University, Ithaca, NY 14853, USA
[5]Boyce Thompson Institute, Ithaca, NY 14853, USA
*corresponding author: marek.elias@osu.cz

Supplementary Dataset S2. Gene maps of the newly generated eustigmatophyte mitochondrial genomes. The display convention is the same as in Dataset. S1.

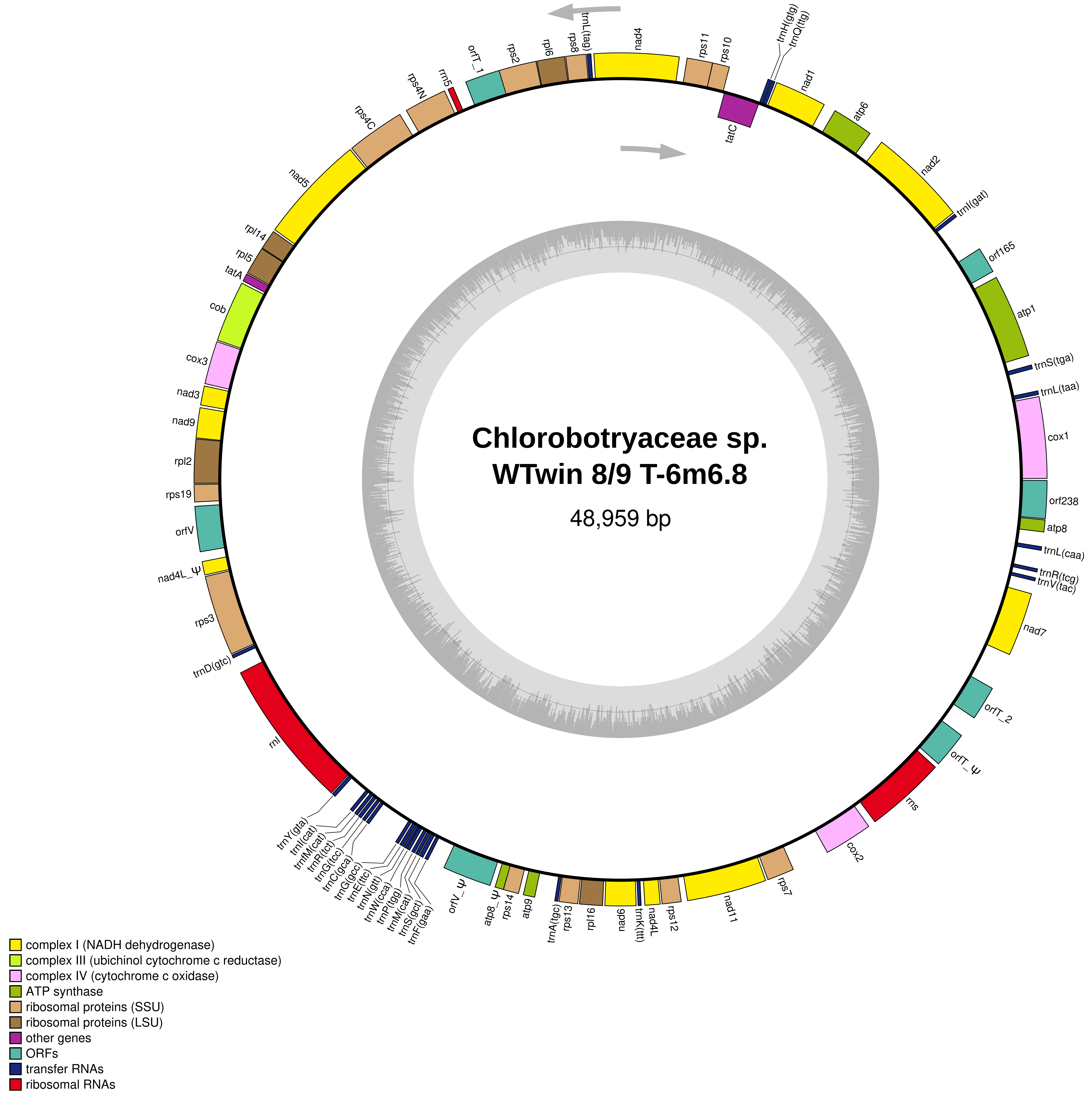
Chlorobotryaceae sp.
WTwin 8/9 T-6m6.8
48,959 bp
nad4
rps11
rps10
tatC
trnH(gtg)
trnQ(ttg)
nad1
atp6
nad2
trnI(gat)
orf165
atp1
trnS(tga)
trnL(taa)
cox1
orf238
atp8
trnL(caa)
trnR(tcg)
trnV(tac)
nad7
orfT_2
orfT_Ψ
rns
cox2
rps7
nad11
rps12
nad4L
trnK(ttt)
nad6
rpl16
rps13
trnA(tgc)
atp9
rps14
atp8_Ψ
orfV_Ψ
trnF(gaa)
trnS(gct)
trnM(cat)
trnP(tgg)
trnW(cca)
trnN(gtt)
trnE(ttc)
trnG(gcc)
trnC(gca)
trnG(tcc)
trnR(tct)
trnfM(cat)
trnI(cat)
trnY(gta)
rnl
trnD(gtc)
rps3
nad4L_Ψ
orfV
rps19
rpl2
nad9
nad3
cox3
cob
tatA
rpl5
rpl14
nad5
rps4C
rps4N
rrn5
orfT_1
rps2
rpl6
rps8
trnL(tag)
complex I (NADH dehydrogenase)
complex III (ubichinol cytochrome c reductase)
complex IV (cytochrome c oxidase)
ATP synthase
ribosomal proteins (SSU)
ribosomal proteins (LSU)
other genes
ORFs
transfer RNAs
ribosomal RNAs

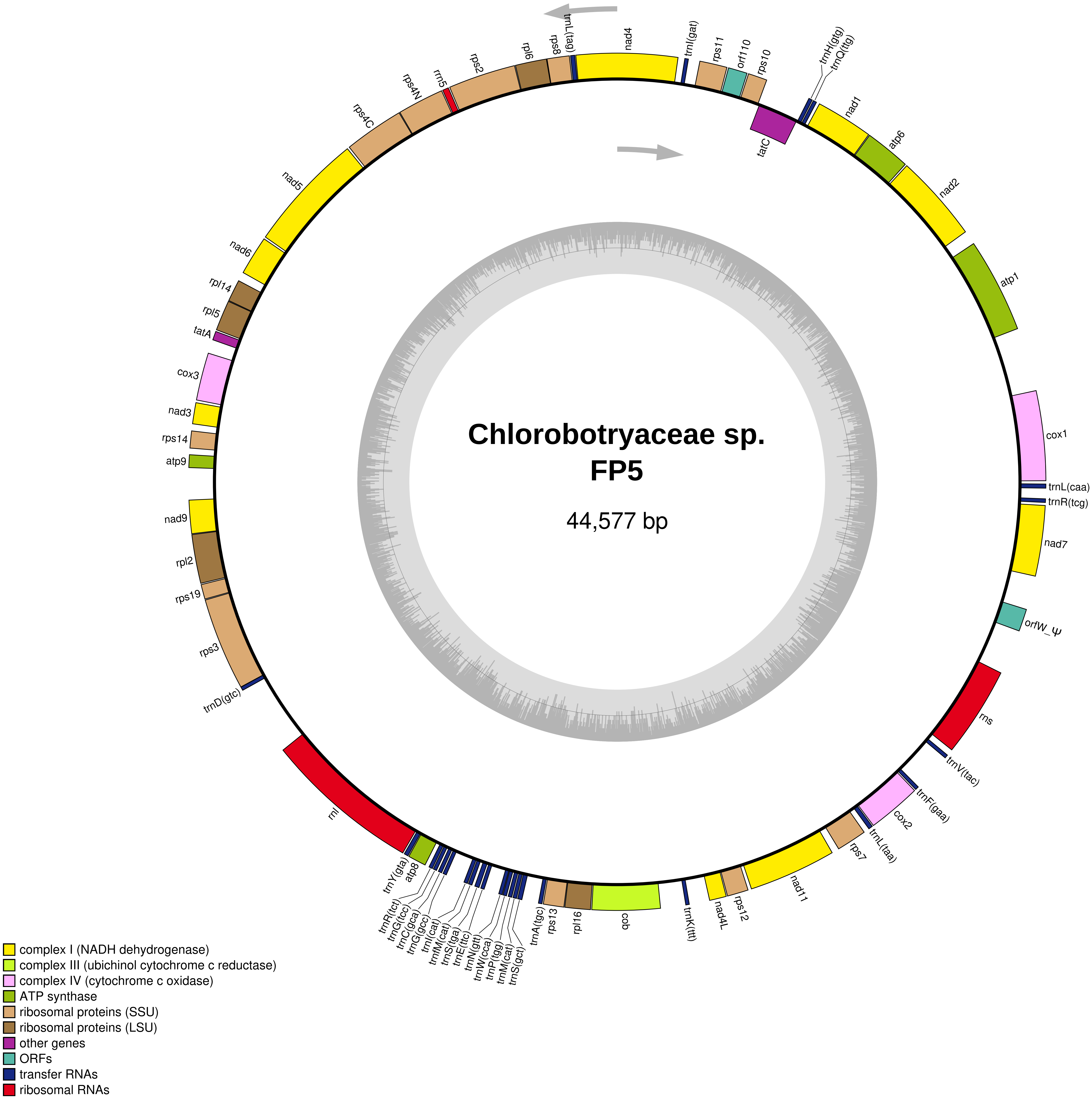

Chlorobotryaceae sp. FP5
44,577 bp
nad4
trnI(gat)
rps11
orf110
rps10
trnH(gtg)
trnQ(ttg)
tatC
nad1
atp6
nad2
atp1
cox1
trnL(caa)
trnR(tcg)
nad7
orfW_Ψ
rns
trnV(tac)
trnF(gaa)
cox2
trnL(taa)
rps7
nad11
rps12
nad4L
trnK(ttt)
cob
rpl16
rps13
trnA(tgc)
trnS(gct)
trnM(cat)
trnP(tgg)
trnW(cca)
trnN(gtt)
trnE(ttc)
trnS(tga)
trnfM(cat)
trnI(cat)
trnG(gcc)
trnC(gca)
trnG(tcc)
trnR(tct)
atp8
trnY(gta)
rnl
trnD(gtc)
rps3
rps19
rpl2
nad9
atp9
rps14
nad3
cox3
tatA
rpl5
rpl14
nad6
nad5
rps4C
rps4N
rrn5
rps2
rpl6
rps8
trnL(tag)
complex I (NADH dehydrogenase)
complex III (ubichinol cytochrome c reductase)
complex IV (cytochrome c oxidase)
ATP synthase
ribosomal proteins (SSU)
ribosomal proteins (LSU)
other genes
ORFs
transfer RNAs
ribosomal RNAs

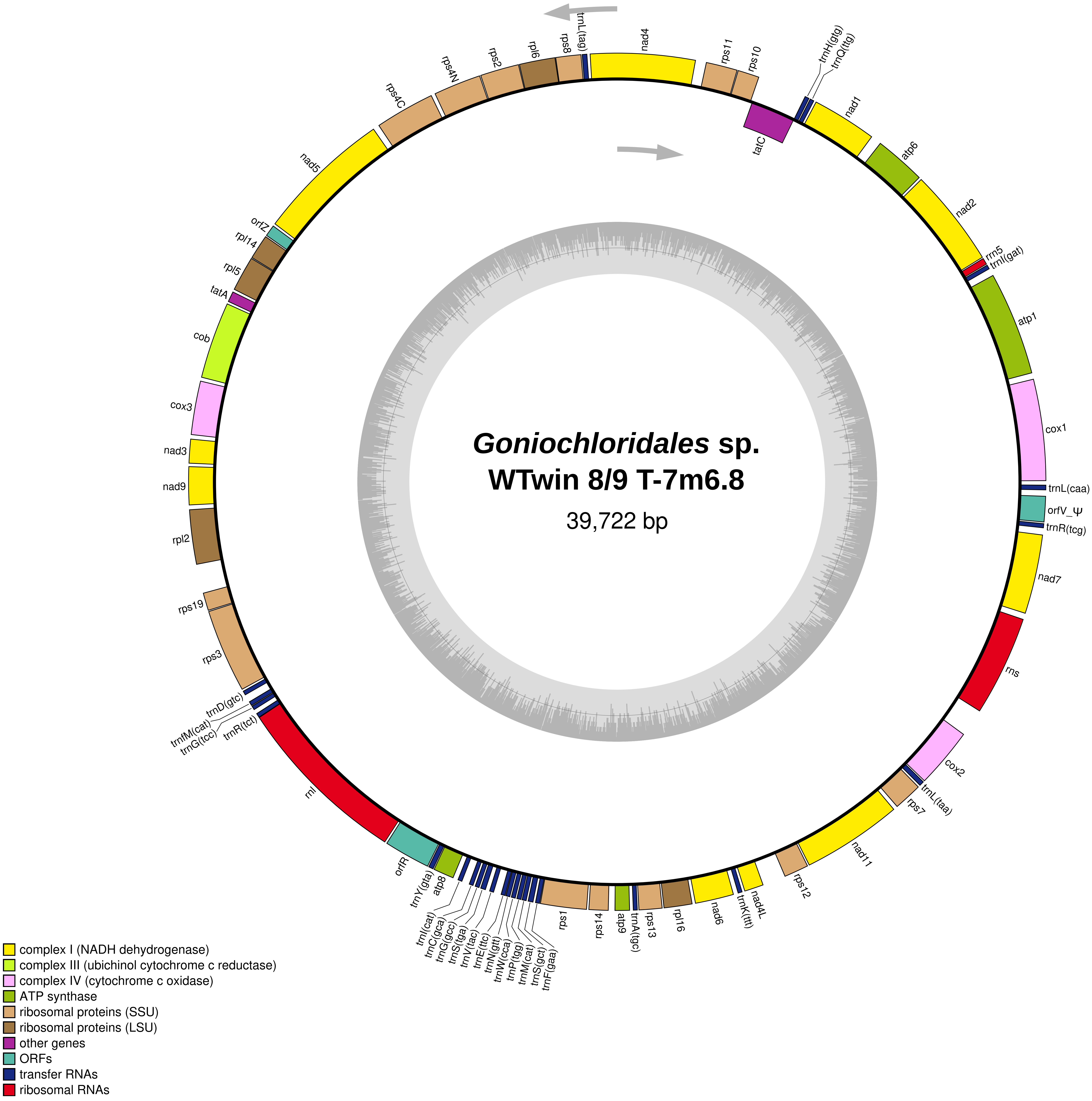
Goniochloridales sp.
WTwin 8/9 T-7m6.8
39,722 bp
nad4
rps11
rps10
trnH(gtg)
trnQ(ttg)
tatC
nad1
atp6
nad2
rrn5
trnI(gat)
atp1
cox1
trnL(caa)
orfV_Ψ
trnR(tcg)
nad7
rns
cox2
trnL(taa)
rps7
nad11
rps12
nad4L
trnK(ttt)
nad6
rpl16
rps13
trnA(tgc)
atp9
rps14
rps1
trnF(gaa)
trnS(gct)
trnM(cat)
trnP(tgg)
trnW(cca)
trnN(gtt)
trnE(ttc)
trnV(tac)
trnS(tga)
trnG(gcc)
trnC(gca)
trnI(cat)
atp8
trnY(gta)
orfR
rnl
trnR(tct)
trnG(tcc)
trnfM(cat)
trnD(gtc)
rps3
rps19
rpl2
nad9
nad3
cox3
cob
tatA
rpl5
rpl14
orfZ
nad5
rps4C
rps4N
rps2
rpl6
rps8
trnL(tag)
complex I (NADH dehydrogenase)
complex III (ubichinol cytochrome c reductase)
complex IV (cytochrome c oxidase)
ATP synthase
ribosomal proteins (SSU)
ribosomal proteins (LSU)
other genes
ORFs
transfer RNAs
ribosomal RNAs

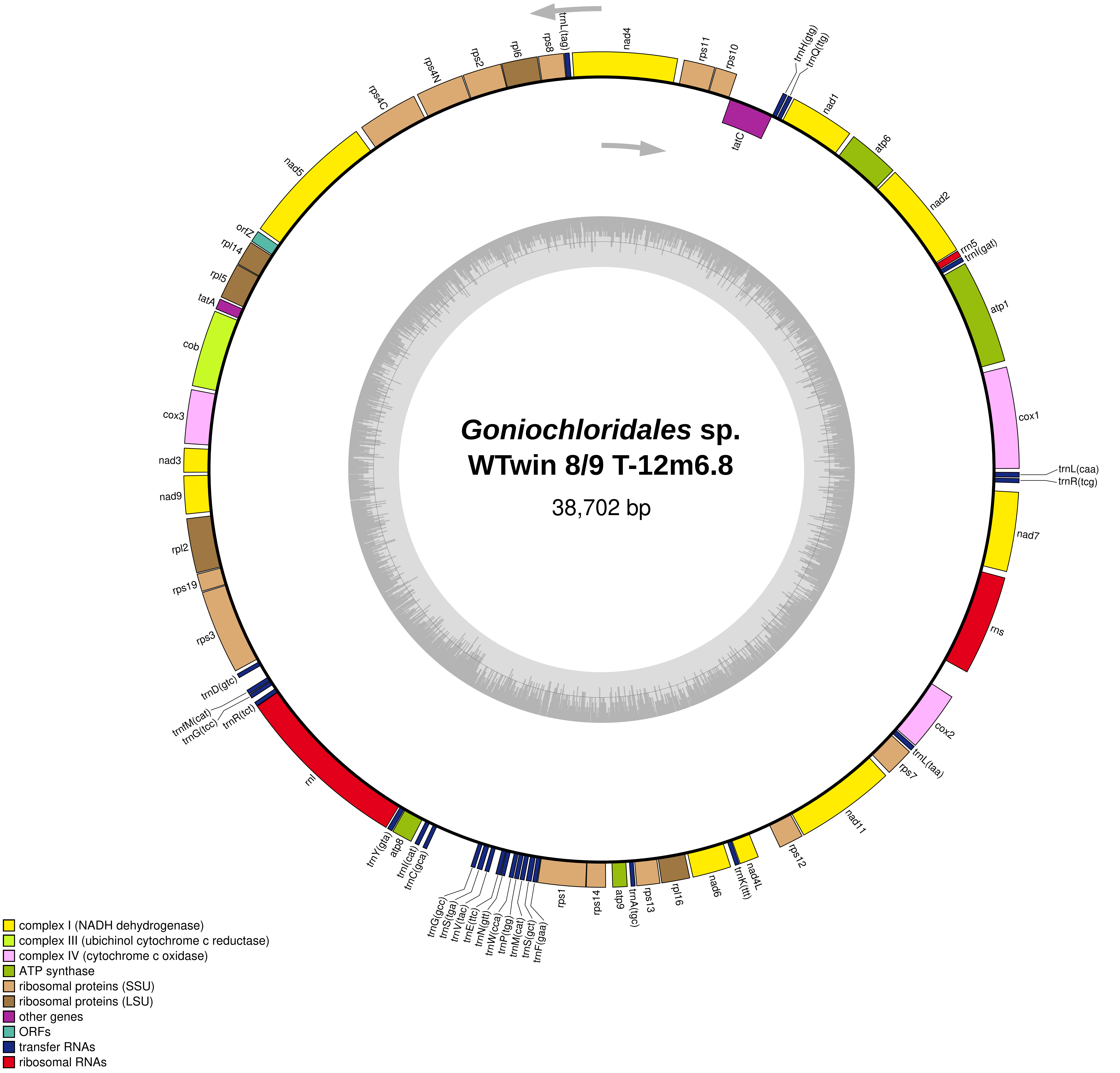

Goniochloridales sp.
WTwin 8/9 T-12m6.8
38,702 bp
trnL(tag)
nad4
rps11
rps10
trnH(gtg)
trnQ(ttg)
tatC
nad1
atp6
nad2
rrn5
trnI(gat)
atp1
cox1
trnL(caa)
trnR(tcg)
nad7
rns
cox2
trnL(taa)
rps7
nad11
rps12
nad4L
trnK(ttt)
nad6
rpl16
rps13
trnA(tgc)
atp9
rps14
rps1
trnG(gcc)
trnS(tga)
trnV(tac)
trnE(ttc)
trnN(gtt)
trnW(cca)
trnP(tgg)
trnM(cat)
trnS(gct)
trnF(gaa)
trnC(gca)
trnI(cat)
atp8
trnY(gta)
rnl
trnR(tct)
trnG(tcc)
trnfM(cat)
trnD(gtc)
rps3
rps19
rpl2
nad9
nad3
cox3
cob
tatA
rpl5
rpl14
orfZ
nad5
rps4C
rps4N
rps2
rpl6
rps8
complex I (NADH dehydrogenase)
complex III (ubichinol cytochrome c reductase)
complex IV (cytochrome c oxidase)
ATP synthase
ribosomal proteins (SSU)
ribosomal proteins (LSU)
other genes
ORFs
transfer RNAs
ribosomal RNAs

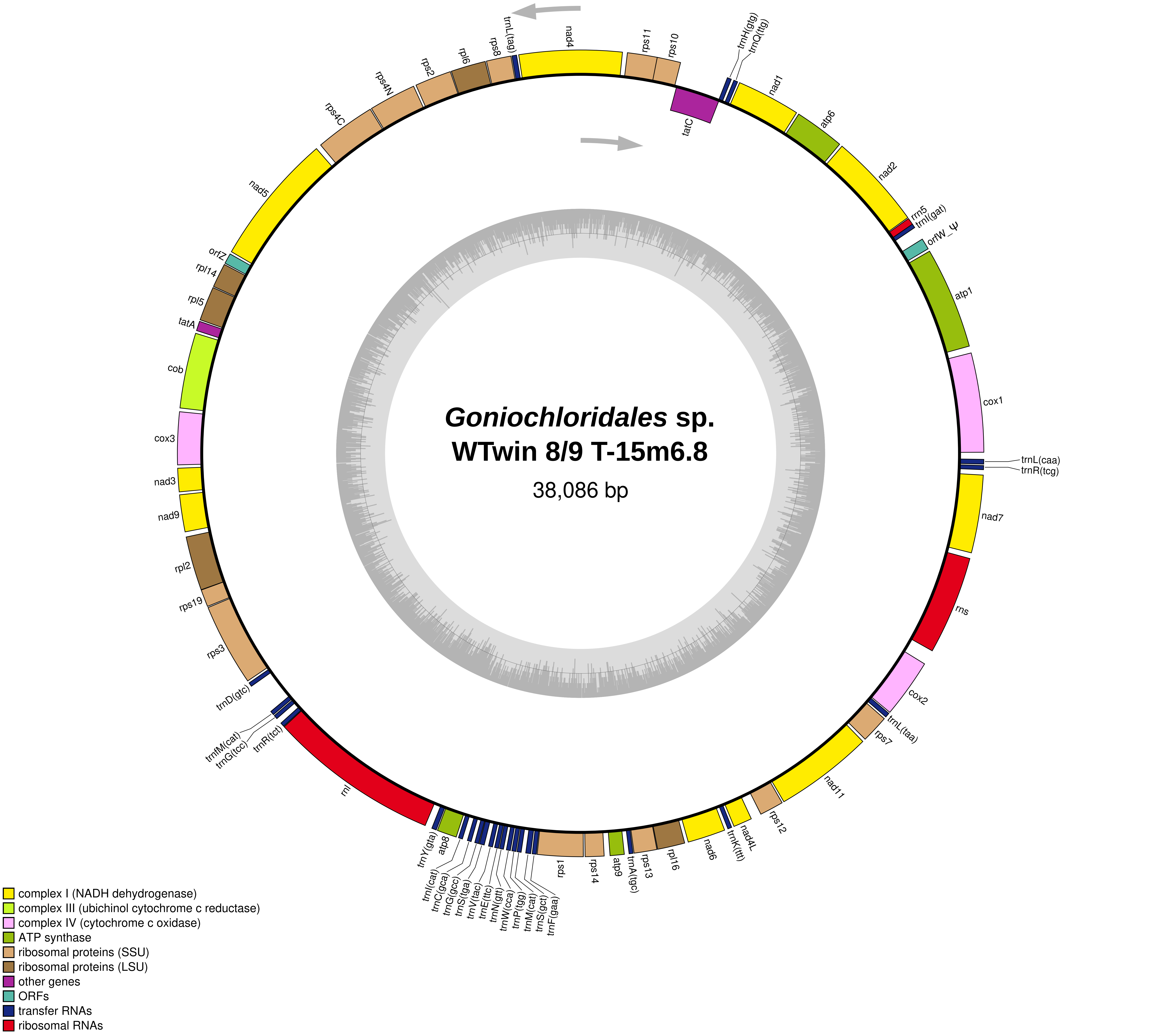
Goniochloridales sp.
WTwin 8/9 T-15m6.8
38,086 bp
nad4
rps11
rps10
tatC
trnH(gtg)
trnQ(ttg)
nad1
atp6
nad2
rrn5
trnI(gat)
orfW_Ψ
atp1
cox1
trnL(caa)
trnR(tcg)
nad7
rns
cox2
trnL(taa)
rps7
nad11
rps12
nad4L
trnK(ttt)
nad6
rpl16
rps13
trnA(tgc)
atp9
rps14
rps1
trnF(gaa)
trnS(gct)
trnM(cat)
trnP(tgg)
trnW(cca)
trnN(gtt)
trnE(ttc)
trnV(tac)
trnS(tga)
trnG(gcc)
trnC(gca)
trnI(cat)
atp8
trnY(gta)
rnl
trnR(tct)
trnG(tcc)
trnfM(cat)
trnD(gtc)
rps3
rps19
rpl2
nad9
nad3
cox3
cob
tatA
rpl5
rpl14
orfZ
nad5
rps4C
rps4N
rps2
rpl6
rps8
trnL(tag)
complex I (NADH dehydrogenase)
complex III (ubichinol cytochrome c reductase)
complex IV (cytochrome c oxidase)
ATP synthase
ribosomal proteins (SSU)
ribosomal proteins (LSU)
other genes
ORFs
transfer RNAs
ribosomal RNAs

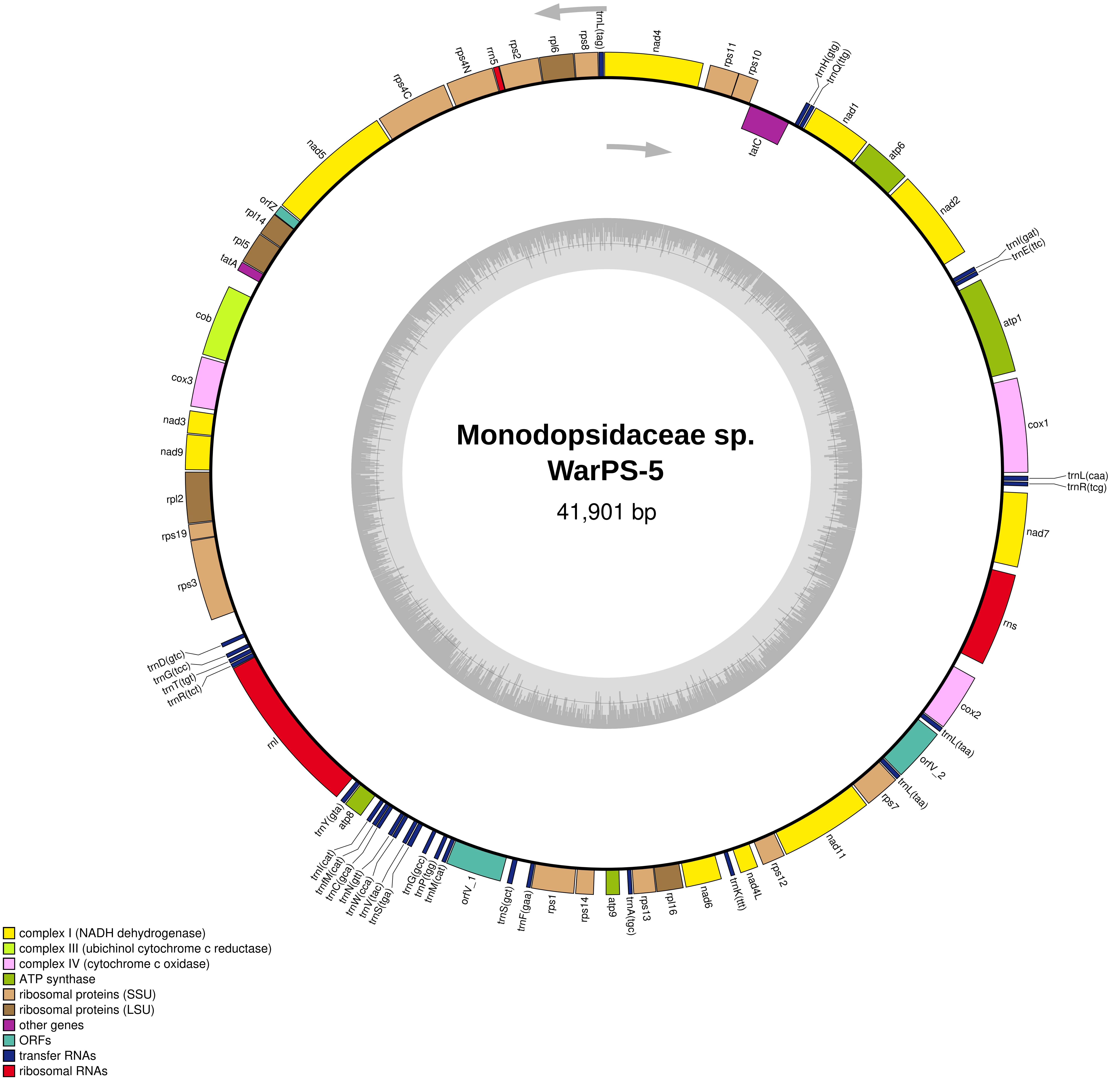
Monodopsidaceae sp.
WarPS-5
41,901 bp
trnL(tag)
nad4
rps11
rps10
trnH(gtg)
trnQ(ttg)
tatC
nad1
atp6
nad2
trnI(gat)
trnE(ttc)
atp1
cox1
trnL(caa)
trnR(tcg)
nad7
rns
cox2
trnL(taa)
orfV_2
trnL(taa)
rps7
nad11
rps12
nad4L
trnK(ttt)
nad6
rpl16
rps13
trnA(tgc)
atp9
rps14
rps1
trnF(gaa)
trnS(gct)
orfV_1
trnM(cat)
trnP(tgg)
trnG(gcc)
trnS(tga)
trnV(tac)
trnW(cca)
trnN(gtt)
trnC(gca)
trnfM(cat)
trnI(cat)
atp8
trnY(gta)
rnl
trnD(gtc)
trnG(tcc)
trnT(tgt)
trnR(tct)
rps3
rps19
rpl2
nad9
nad3
cox3
cob
tatA
rpl5
rpl14
orfZ
nad5
rps4C
rps4N
rrn5
rps2
rpl6
rps8
complex I (NADH dehydrogenase)
complex III (ubichinol cytochrome c reductase)
complex IV (cytochrome c oxidase)
ATP synthase
ribosomal proteins (SSU)
ribosomal proteins (LSU)
other genes
ORFs
transfer RNAs
ribosomal RNAs

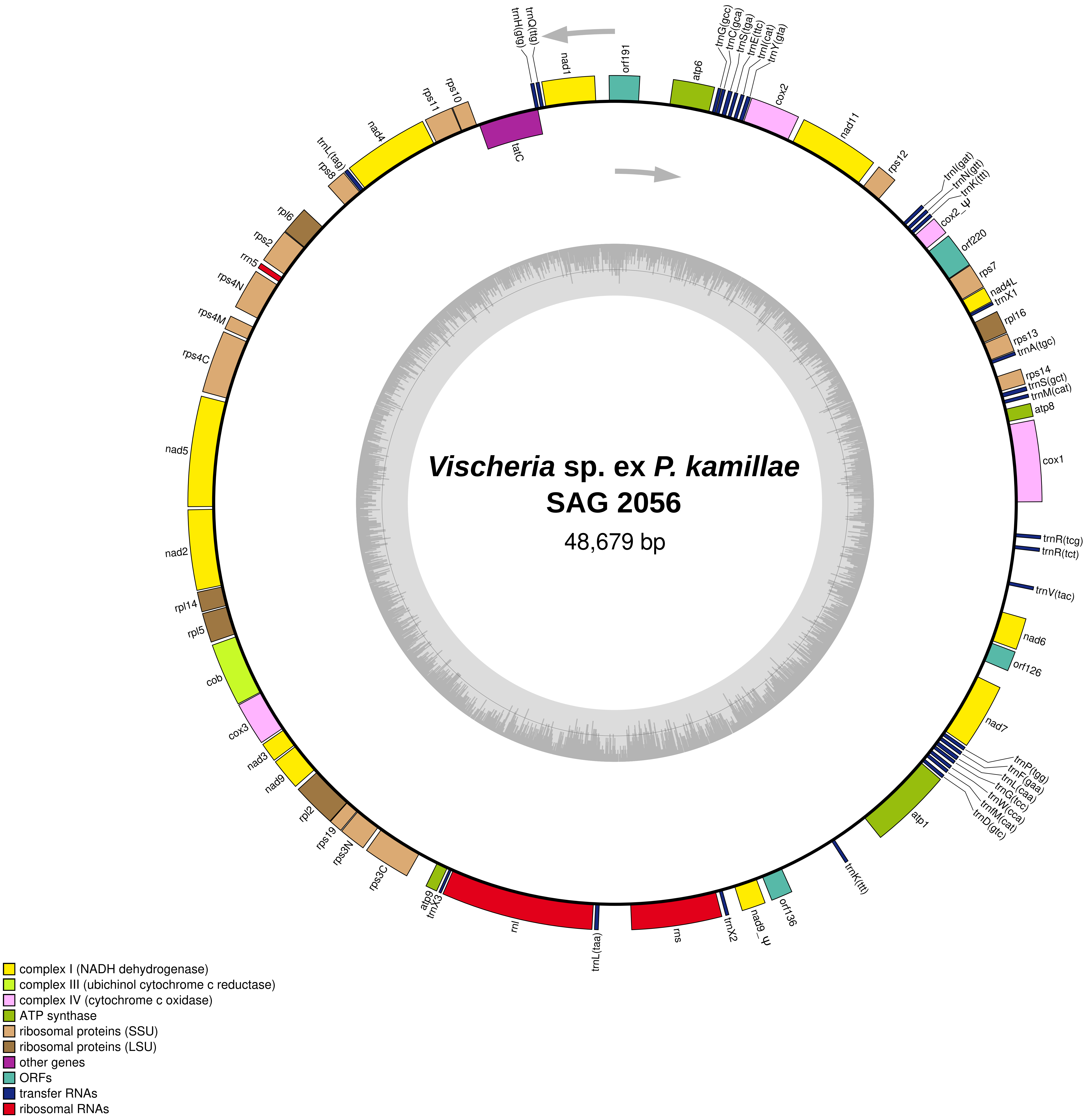

Vischeria sp. ex P. kamillae
SAG 2056
48,679 bp
trnH(gtg)
trnQ(ttg)
nad1
orf191
atp6
trnG(gcc)
trnC(gca)
trnS(tga)
trnE(ttc)
trnI(cat)
trnY(gta)
cox2
nad11
rps12
trnI(gat)
trnN(gtt)
trnK(ttt)
cox2_ψ
orf220
rps7
nad4L
trnX1
rpl16
rps13
trnA(tgc)
rps14
trnS(gct)
trnM(cat)
atp8
cox1
trnR(tcg)
trnR(tct)
trnV(tac)
nad6
orf126
nad7
trnP(tgg)
trnF(gaa)
trnL(caa)
trnG(tcc)
trnW(cca)
trnM(cat)
trnD(gtc)
atp1
trnK(ttt)
orf136
nad9_ψ
trnX2
rns
trnL(taa)
rnl
trnX3
atp9
rps3C
rps3N
rps19
rpl2
nad9
nad3
cox3
cob
rpl5
rpl14
nad2
nad5
rps4C
rps4M
rps4N
rrn5
rps2
rpl6
rps8
trnL(tag)
nad4
rps11
rps10
tatC
complex I (NADH dehydrogenase)
complex III (ubichinol cytochrome c reductase)
complex IV (cytochrome c oxidase)
ATP synthase
ribosomal proteins (SSU)
ribosomal proteins (LSU)
other genes
ORFs
transfer RNAs
ribosomal RNAs

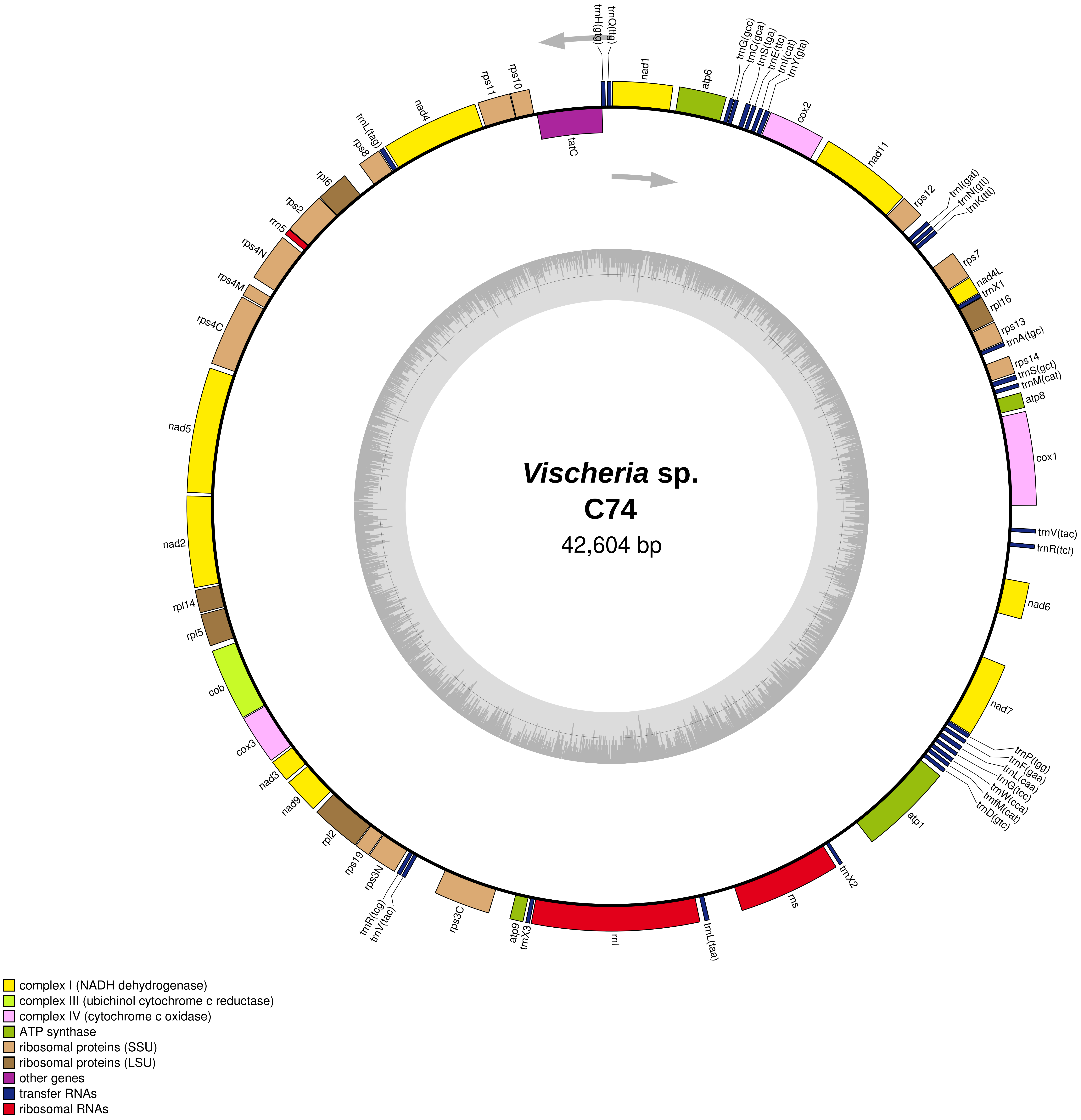

Vischeria sp.
C74
42,604 bp
trnH(gtg)
trnQ(ttg)
nad1
atp6
trnG(gcc)
trnC(gca)
trnS(tga)
trnE(ttc)
trnI(cat)
trnY(gta)
cox2
nad11
rps12
trnI(gat)
trnN(gtt)
trnK(ttt)
rps7
nad4L
trnX1
rpl16
rps13
trnA(tgc)
rps14
trnS(gct)
trnM(cat)
atp8
cox1
trnV(tac)
trnR(tct)
nad6
nad7
trnP(tgg)
trnF(gaa)
trnL(caa)
trnG(tcc)
trnW(cca)
trnfM(cat)
trnD(gtc)
atp1
trnX2
rns
trnL(taa)
rnl
trnX3
atp9
rps3C
trnV(tac)
trnR(tcg)
rps3N
rps19
rpl2
nad9
nad3
cox3
cob
rpl5
rpl14
nad2
nad5
rps4C
rps4M
rps4N
rrn5
rps2
rpl6
rps8
trnL(tag)
nad4
rps11
rps10
tatC
complex I (NADH dehydrogenase)
complex III (ubichinol cytochrome c reductase)
complex IV (cytochrome c oxidase)
ATP synthase
ribosomal proteins (SSU)
ribosomal proteins (LSU)
other genes
transfer RNAs
ribosomal RNAs

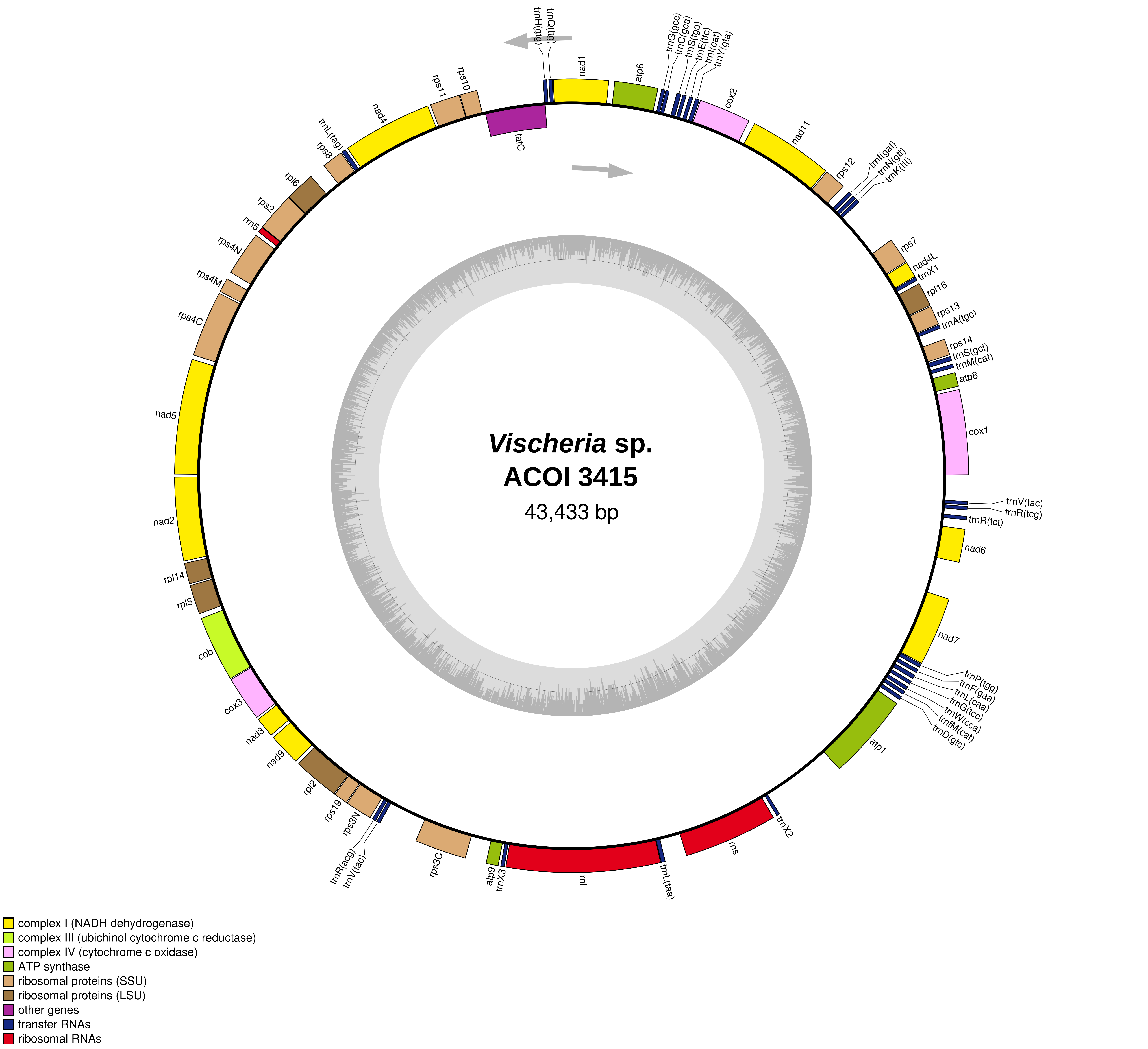

Vischeria sp.
ACOI 3415
43,433 bp
trnH(gtg)
trnQ(ttg)
nad1
atp6
trnG(gcc)
trnC(gca)
trnS(tga)
trnE(ttc)
trnI(cat)
trnY(gta)
cox2
nad11
rps12
trnI(gat)
trnN(gtt)
trnK(ttt)
rps7
nad4L
trnX1
rpl16
rps13
trnA(tgc)
rps14
trnS(gct)
trnM(cat)
atp8
cox1
trnV(tac)
trnR(tcg)
trnR(tct)
nad6
nad7
trnP(tgg)
trnF(gaa)
trnL(caa)
trnG(tcc)
trnW(cca)
trnfM(cat)
trnD(gtc)
atp1
trnX2
rns
trnL(taa)
rnl
trnX3
atp9
rps3C
trnV(tac)
trnR(acg)
rps3N
rps19
rpl2
nad9
nad3
cox3
cob
rpl5
rpl14
nad2
nad5
rps4C
rps4M
rps4N
rrn5
rps2
rpl6
rps8
trnL(tag)
nad4
rps11
rps10
tatC
complex I (NADH dehydrogenase)
complex III (ubichinol cytochrome c reductase)
complex IV (cytochrome c oxidase)
ATP synthase
ribosomal proteins (SSU)
ribosomal proteins (LSU)
other genes
transfer RNAs
ribosomal RNAs

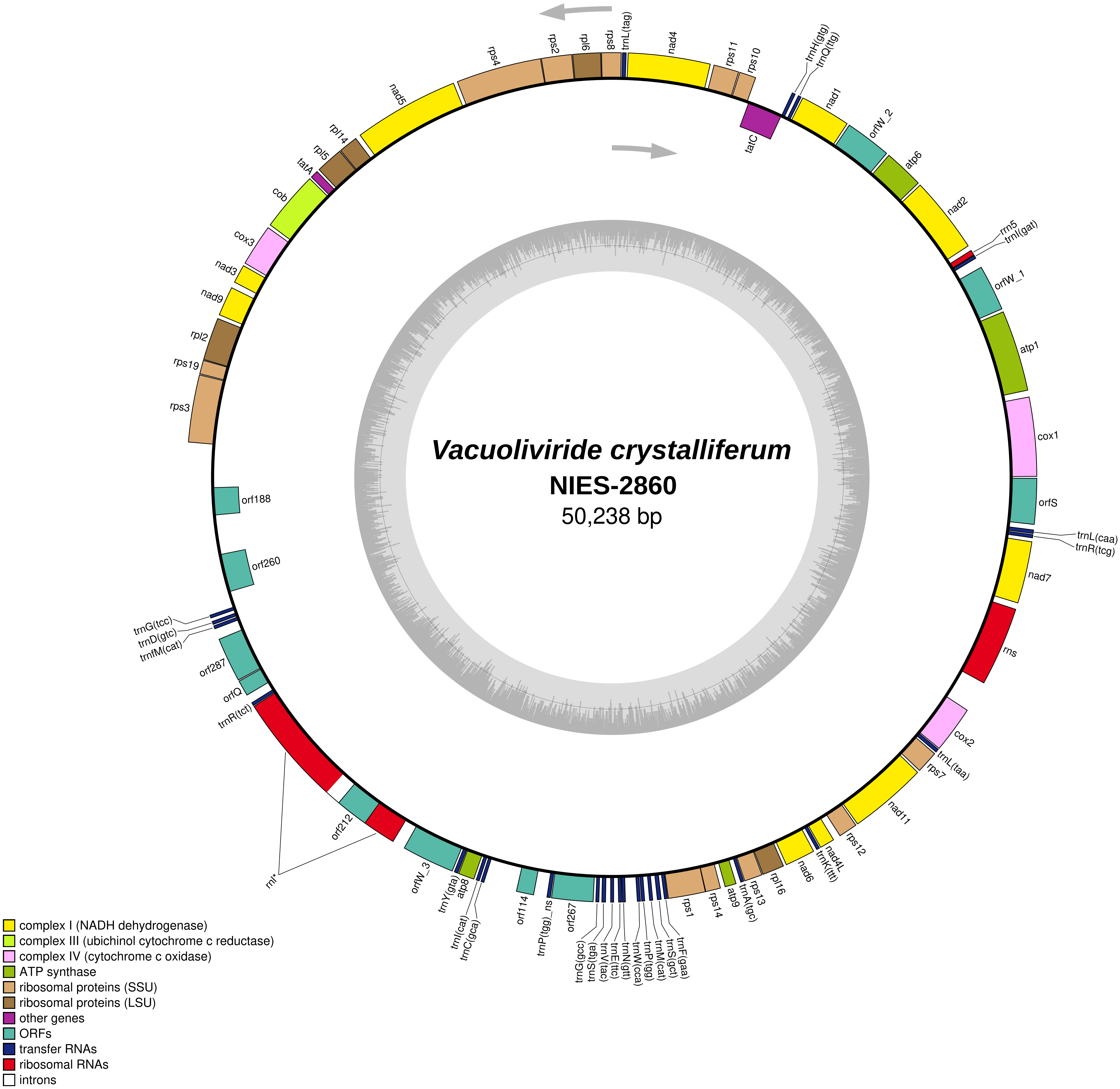

Vacuoliviride crystalliferum
NIES-2860
50,238 bp
trnL(tag)
nad4
rps11
rps10
trnH(gtg)
trnQ(ttg)
tatC
nad1
orfW_2
atp6
nad2
rrn5
trnI(gat)
orfW_1
atp1
cox1
orfS
trnL(caa)
trnR(tcg)
nad7
rns
cox2
trnL(taa)
rps7
nad11
rps12
nad4L
trnK(ttt)
nad6
rpl16
rps13
trnA(tgc)
atp9
rps14
rps1
trnF(gaa)
trnS(gct)
trnM(cat)
trnP(tgg)
trnW(cca)
trnN(gtt)
trnE(ttc)
trnV(tac)
trnS(tga)
trnG(gcc)
orf267
trnP(tgg)_ns
orf114
trnC(gca)
trnI(cat)
atp8
trnY(gta)
orfW_3
rnl*
orf212
trnR(tct)
orfQ
orf287
trnfM(cat)
trnD(gtc)
trnG(tcc)
orf260
orf188
rps3
rps19
rpl2
nad9
nad3
cox3
cob
tatA
rpl5
rpl14
nad5
rps4
rps2
rpl6
rps8
complex I (NADH dehydrogenase)
complex III (ubichinol cytochrome c reductase)
complex IV (cytochrome c oxidase)
ATP synthase
ribosomal proteins (SSU)
ribosomal proteins (LSU)
other genes
ORFs
transfer RNAs
ribosomal RNAs
introns

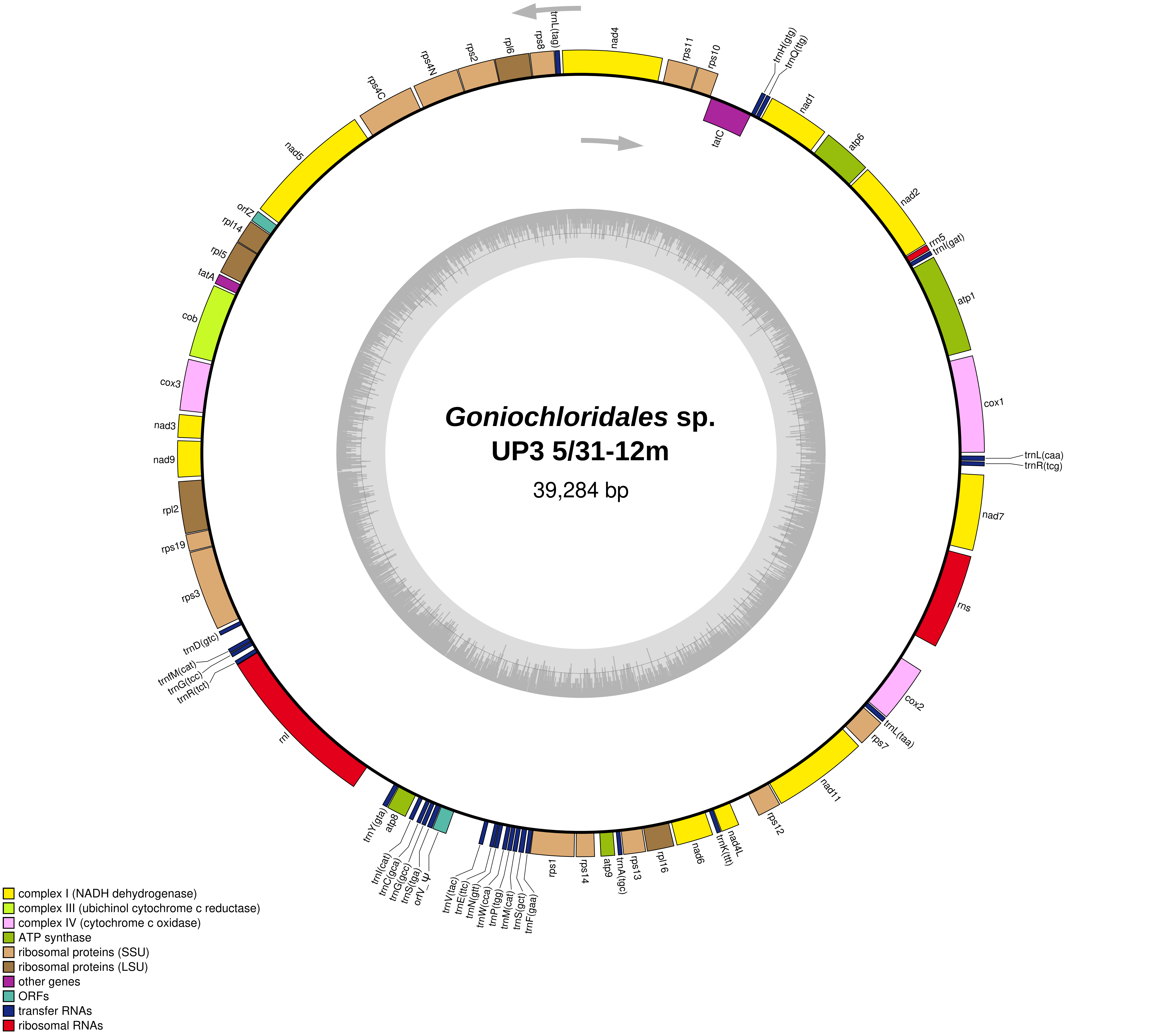
Goniochloridales sp.
UP3 5/31-12m
39,284 bp
trnL(tag)
nad4
rps11
rps10
trnH(gtg)
trnQ(ttg)
tatC
nad1
atp6
nad2
rrn5
trnI(gat)
atp1
cox1
trnL(caa)
trnR(tcg)
nad7
rns
cox2
trnL(taa)
rps7
nad11
rps12
nad4L
trnK(ttt)
nad6
rpl16
rps13
trnA(tgc)
atp9
rps14
rps1
trnV(tac)
trnE(ttc)
trnN(gtt)
trnW(cca)
trnP(tgg)
trnM(cat)
trnS(gct)
trnF(gaa)
trnI(cat)
trnC(gca)
trnG(gcc)
trnS(tga)
orfV_ψ
trnY(gta)
atp8
rnl
trnfM(cat)
trnG(tcc)
trnR(tct)
trnD(gtc)
rps3
rps19
rpl2
nad9
nad3
cox3
cob
tatA
rpl5
rpl14
orfZ
nad5
rps4C
rps4N
rps2
rpl6
rps8
complex I (NADH dehydrogenase)
complex III (ubichinol cytochrome c reductase)
complex IV (cytochrome c oxidase)
ATP synthase
ribosomal proteins (SSU)
ribosomal proteins (LSU)
other genes
ORFs
transfer RNAs
ribosomal RNAs

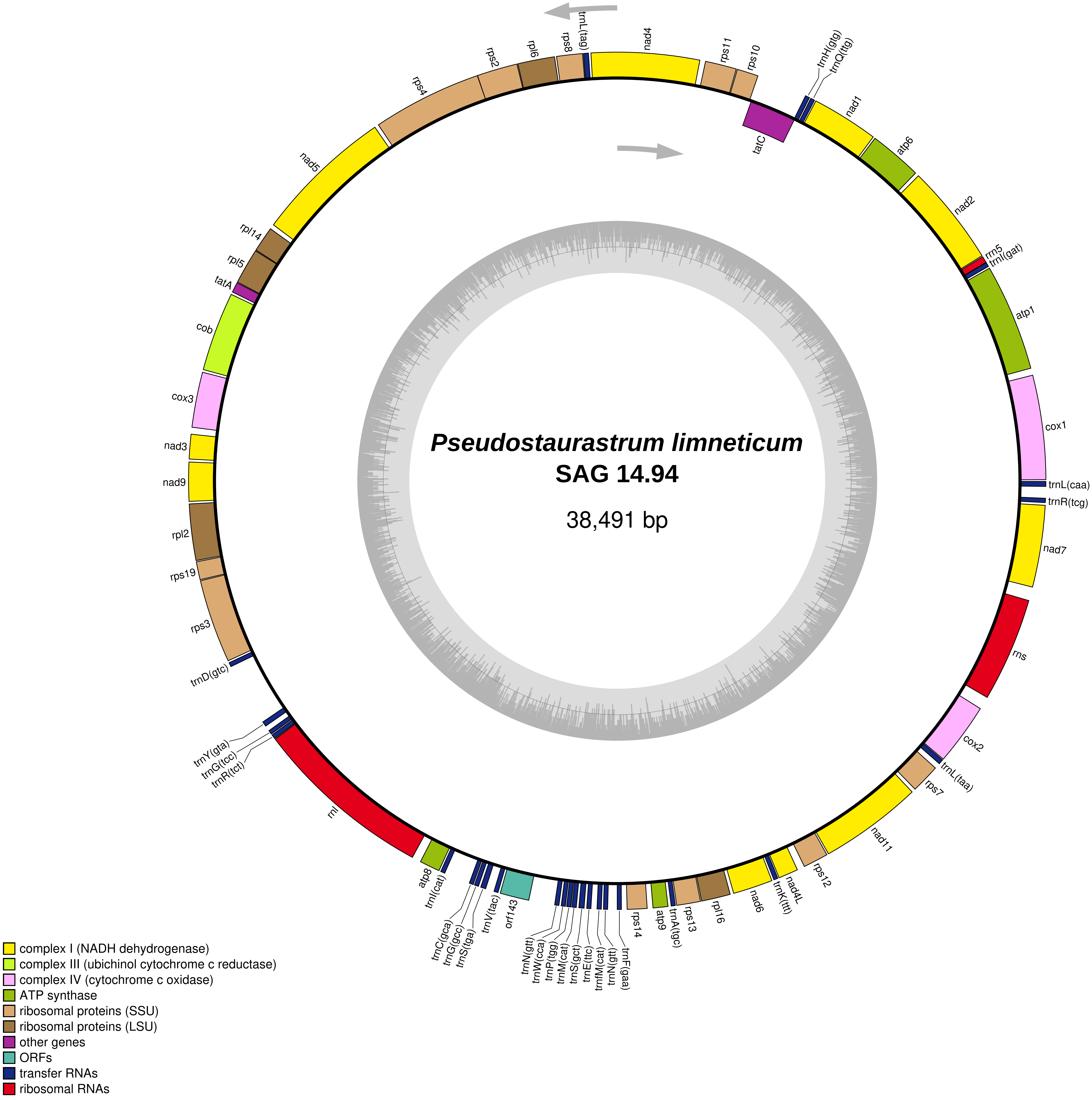
Pseudostaurastrum limneticum
SAG 14.94
38,491 bp
nad4
rps11
rps10
trnH(gtg)
trnQ(ttg)
tatC
nad1
atp6
nad2
rrn5
trnI(gat)
atp1
cox1
trnL(caa)
trnR(tcg)
nad7
rns
cox2
trnL(taa)
rps7
nad11
rps12
nad4L
trnK(ttt)
nad6
rpl16
rps13
trnA(tgc)
atp9
rps14
trnF(gaa)
trnN(gtt)
trnfM(cat)
trnE(ttc)
trnS(gct)
trnM(cat)
trnP(tgg)
trnW(cca)
trnN(gtt)
orf143
trnV(tac)
trnS(tga)
trnG(gcc)
trnC(gca)
trnI(cat)
atp8
rnl
trnR(tct)
trnG(tcc)
trnY(gta)
trnD(gtc)
rps3
rps19
rpl2
nad9
nad3
cox3
cob
tatA
rpl5
rpl14
nad5
rps4
rps2
rpl6
rps8
trnL(tag)
complex I (NADH dehydrogenase)
complex III (ubichinol cytochrome c reductase)
complex IV (cytochrome c oxidase)
ATP synthase
ribosomal proteins (SSU)
ribosomal proteins (LSU)
other genes
ORFs
transfer RNAs
ribosomal RNAs

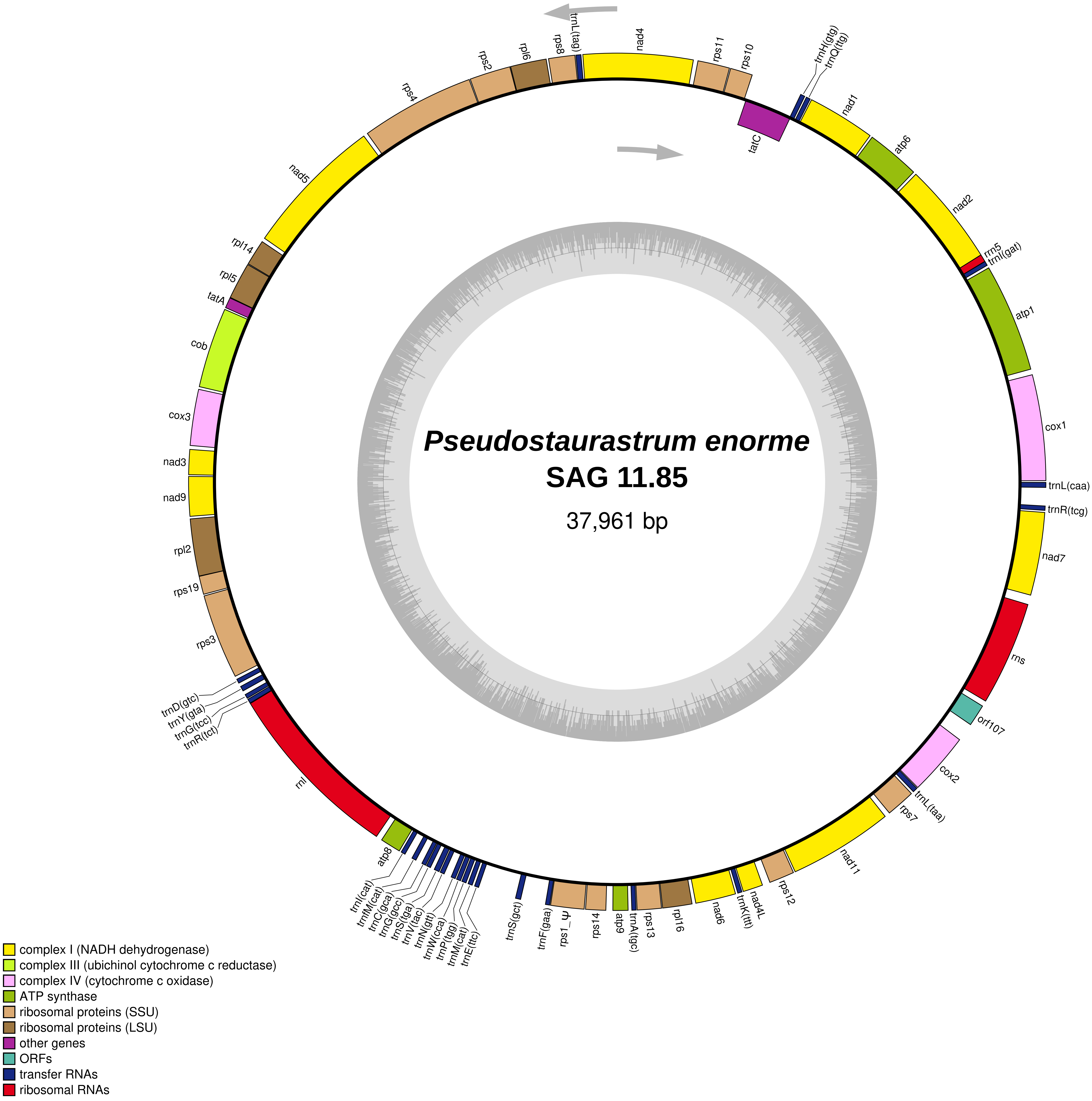

Pseudostaurastrum enorme
SAG 11.85
37,961 bp
nad4
rps11
rps10
trnH(gtg)
trnQ(ttg)
tatC
nad1
atp6
nad2
rrn5
trnI(gat)
atp1
cox1
trnL(caa)
trnR(tcg)
nad7
rns
orf107
cox2
trnL(taa)
rps7
nad11
rps12
nad4L
trnK(ttt)
nad6
rpl16
rps13
trnA(tgc)
atp9
rps14
rps1_ψ
trnF(gaa)
trnS(gct)
trnE(ttc)
trnM(cat)
trnP(tgg)
trnW(cca)
trnN(gtt)
trnV(tac)
trnS(tga)
trnG(gcc)
trnC(gca)
trnfM(cat)
trnI(cat)
atp8
rnl
trnR(tct)
trnG(tcc)
trnY(gta)
trnD(gtc)
rps3
rps19
rpl2
nad9
nad3
cox3
cob
tatA
rpl5
rpl14
nad5
rps4
rps2
rpl6
rps8
trnL(tag)
complex I (NADH dehydrogenase)
complex III (ubichinol cytochrome c reductase)
complex IV (cytochrome c oxidase)
ATP synthase
ribosomal proteins (SSU)
ribosomal proteins (LSU)
other genes
ORFs
transfer RNAs
ribosomal RNAs

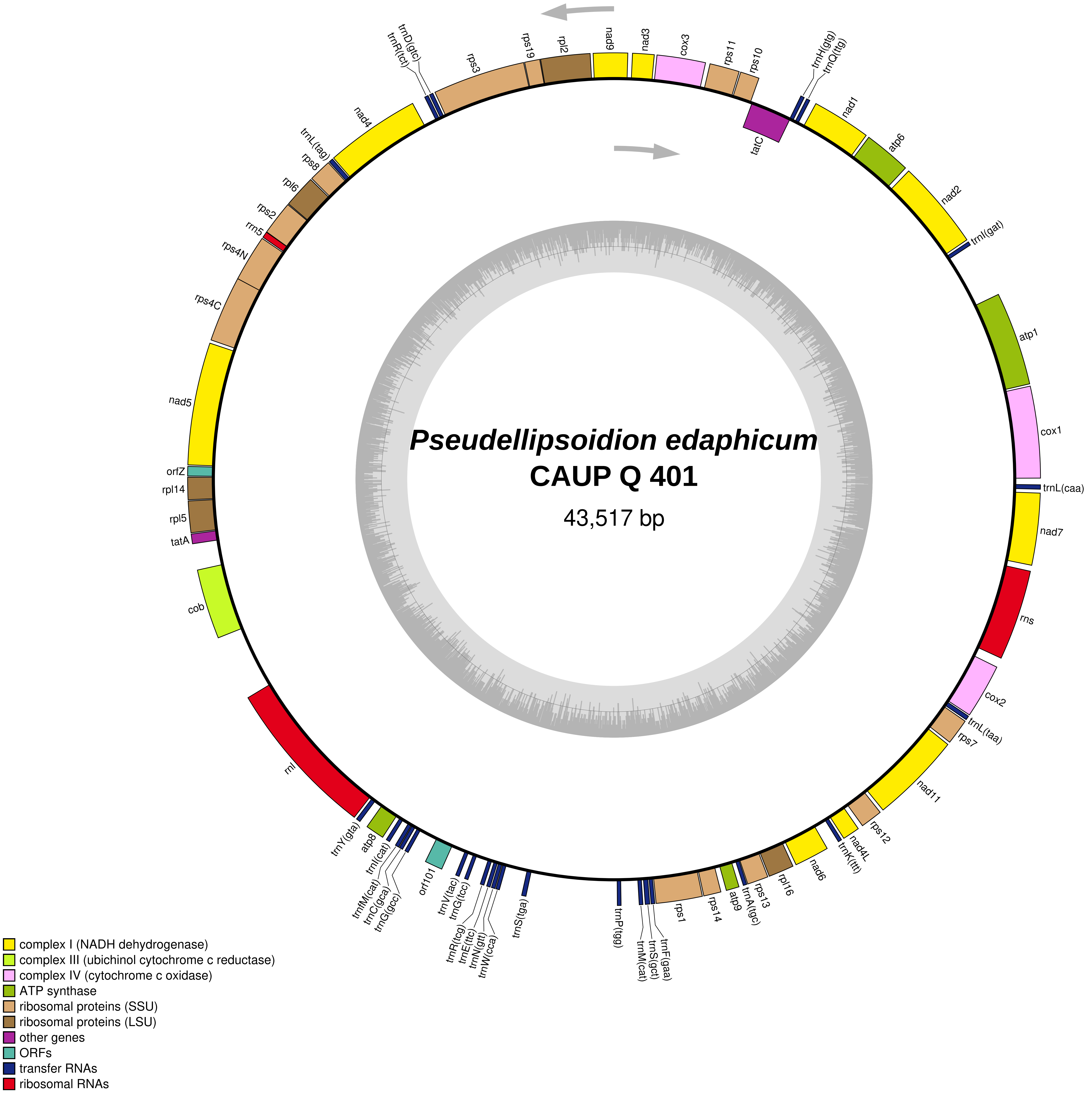
Pseudellipsoidion edaphicum
CAUP Q 401
43,517 bp
trnD(gtc)
trnR(tct)
rps3
rps19
rpl2
nad9
nad3
cox3
rps11
rps10
trnH(gtg)
trnQ(ttg)
tatC
nad1
atp6
nad2
trnI(gat)
atp1
cox1
trnL(caa)
nad7
rns
cox2
trnL(taa)
rps7
nad11
rps12
nad4L
trnK(ttt)
nad6
rpl16
rps13
trnA(tgc)
atp9
rps14
rps1
trnF(gaa)
trnS(gct)
trnM(cat)
trnP(tgg)
trnS(tga)
trnW(cca)
trnN(gtt)
trnE(ttc)
trnR(tcg)
trnG(tcc)
trnV(tac)
orf101
trnG(gcc)
trnC(gca)
trnfM(cat)
trnI(cat)
atp8
trnY(gta)
rnl
cob
tatA
rpl5
rpl14
orfZ
nad5
rps4C
rps4N
rrn5
rps2
rpl6
rps8
trnL(tag)
nad4
complex I (NADH dehydrogenase)
complex III (ubichinol cytochrome c reductase)
complex IV (cytochrome c oxidase)
ATP synthase
ribosomal proteins (SSU)
ribosomal proteins (LSU)
other genes
ORFs
transfer RNAs
ribosomal RNAs

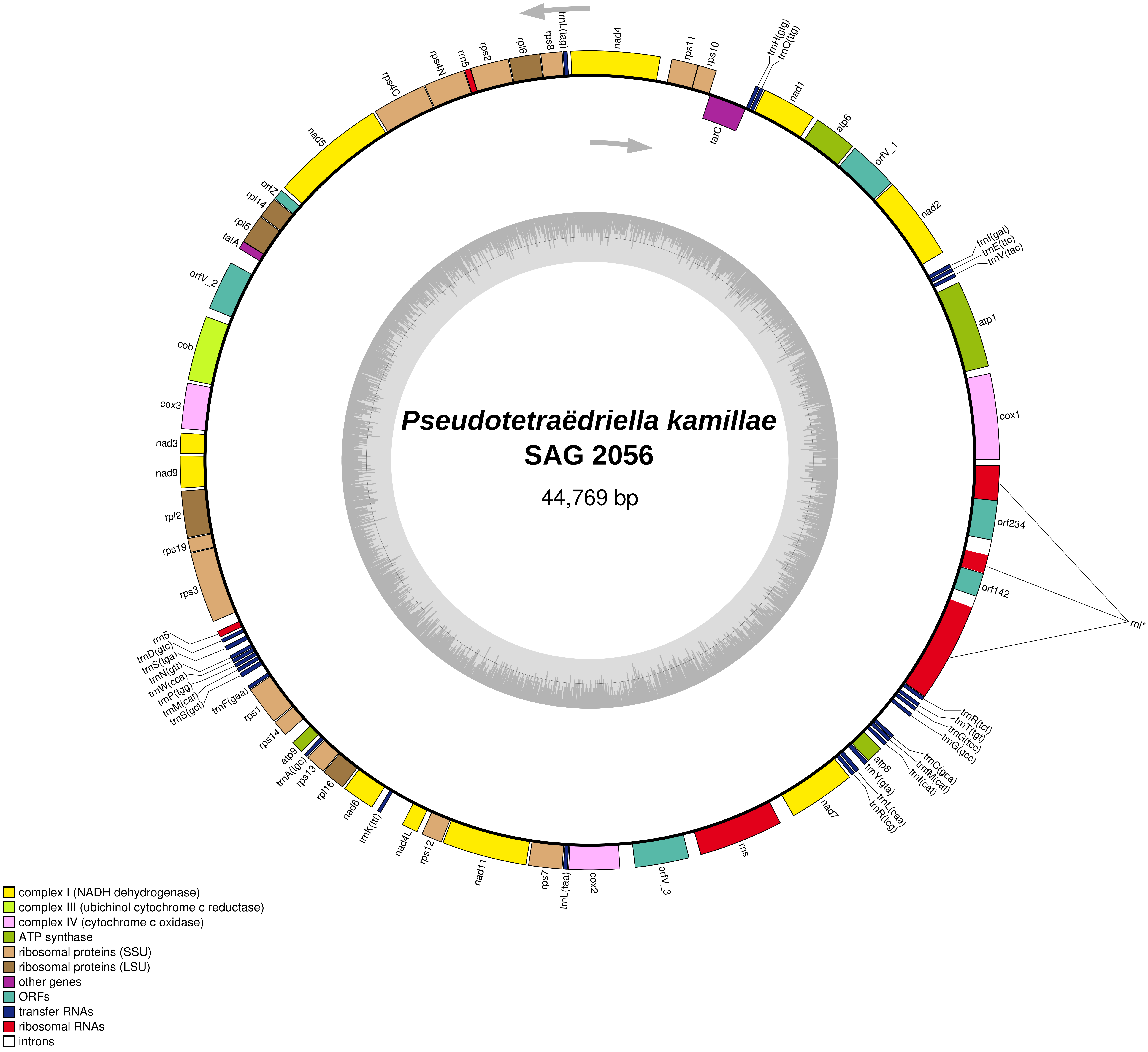

Pseudotetraëdriella kamillae
SAG 2056
44,769 bp
nad4
rps11
rps10
trnH(gtg)
trnQ(ttg)
nad1
atp6
orfV_1
nad2
trnI(gat)
trnE(ttc)
trnV(tac)
atp1
cox1
orf234
orf142
rnl*
trnR(tct)
trnT(tgt)
trnG(tcc)
trnG(gcc)
trnC(gca)
trnfM(cat)
trnI(cat)
atp8
trnY(gta)
trnL(caa)
trnR(tcg)
nad7
rns
orfV_3
cox2
trnL(taa)
rps7
nad11
rps12
nad4L
trnK(ttt)
nad6
rpl16
rps13
trnA(tgc)
atp9
rps14
rps1
trnF(gaa)
trnS(gct)
trnM(cat)
trnP(tgg)
trnW(cca)
trnN(gtt)
trnS(tga)
trnD(gtc)
rrn5
rps3
rps19
rpl2
nad9
nad3
cox3
cob
orfV_2
tatA
rpl5
rpl14
orfZ
nad5
rps4C
rps4N
rrn5
rps2
rpl6
rps8
trnL(tag)
tatC
complex I (NADH dehydrogenase)
complex III (ubichinol cytochrome c reductase)
complex IV (cytochrome c oxidase)
ATP synthase
ribosomal proteins (SSU)
ribosomal proteins (LSU)
other genes
ORFs
transfer RNAs
ribosomal RNAs
introns

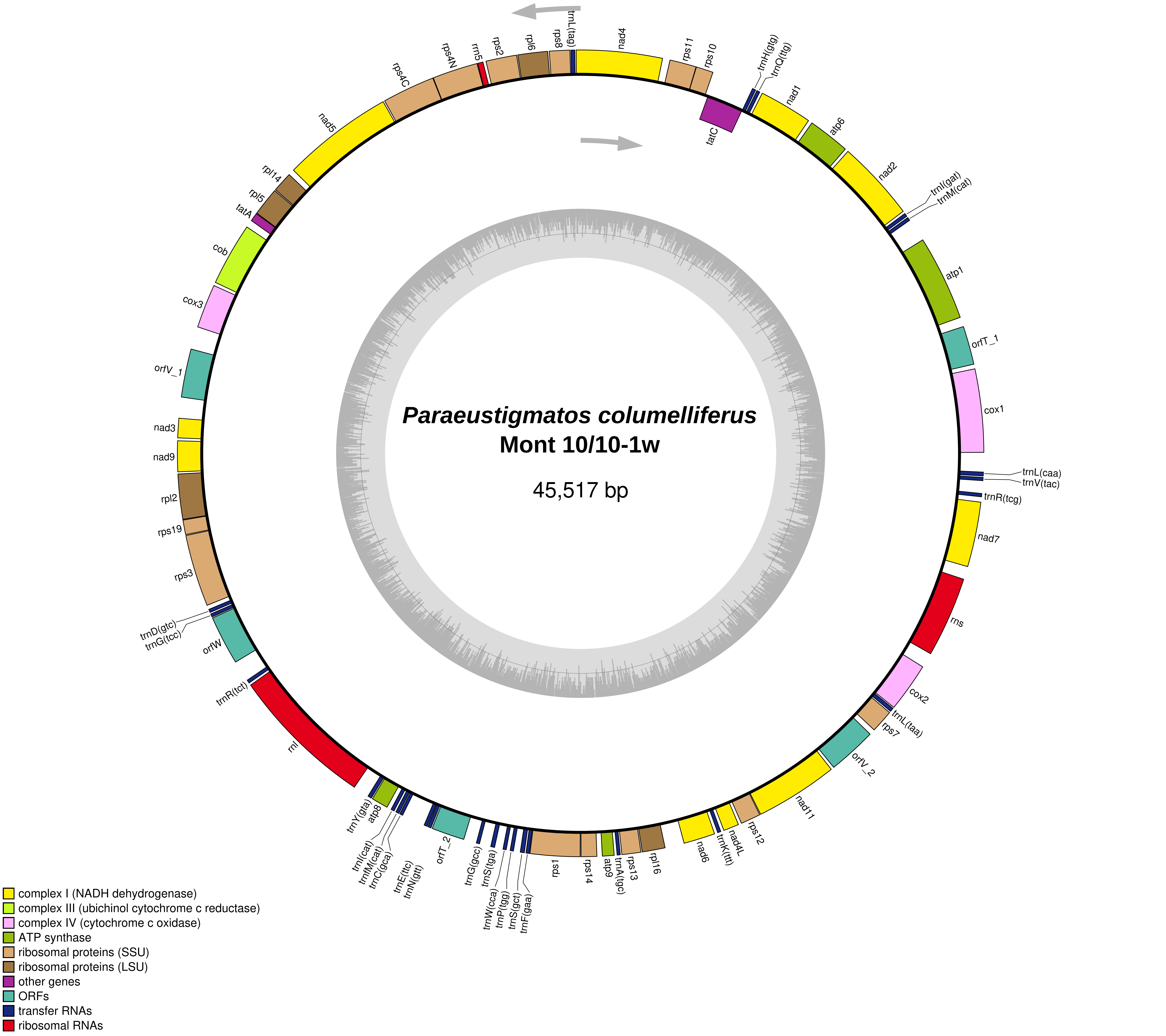
Paraeustigmatos columelliferus
Mont 10/10-1w
45,517 bp
nad4
rps11
rps10
trnH(gtg)
trnQ(ttg)
tatC
nad1
atp6
nad2
trnI(gat)
trnM(cat)
atp1
orfT_1
cox1
trnL(caa)
trnV(tac)
trnR(tcg)
nad7
rns
cox2
trnL(taa)
rps7
orfV_2
nad11
rps12
nad4L
trnK(ttt)
nad6
rpl16
rps13
trnA(tgc)
atp9
rps14
rps1
trnF(gaa)
trnS(gct)
trnP(tgg)
trnW(cca)
trnS(tga)
trnG(gcc)
orfT_2
trnN(gtt)
trnE(ttc)
trnC(gca)
trnfM(cat)
trnI(cat)
atp8
trnY(gta)
rnl
trnR(tct)
orfW
trnG(tcc)
trnD(gtc)
rps3
rps19
rpl2
nad9
nad3
orfV_1
cox3
cob
tatA
rpl5
rpl14
nad5
rps4C
rps4N
rrn5
rps2
rpl6
rps8
trnL(tag)
complex I (NADH dehydrogenase)
complex III (ubichinol cytochrome c reductase)
complex IV (cytochrome c oxidase)
ATP synthase
ribosomal proteins (SSU)
ribosomal proteins (LSU)
other genes
ORFs
transfer RNAs
ribosomal RNAs

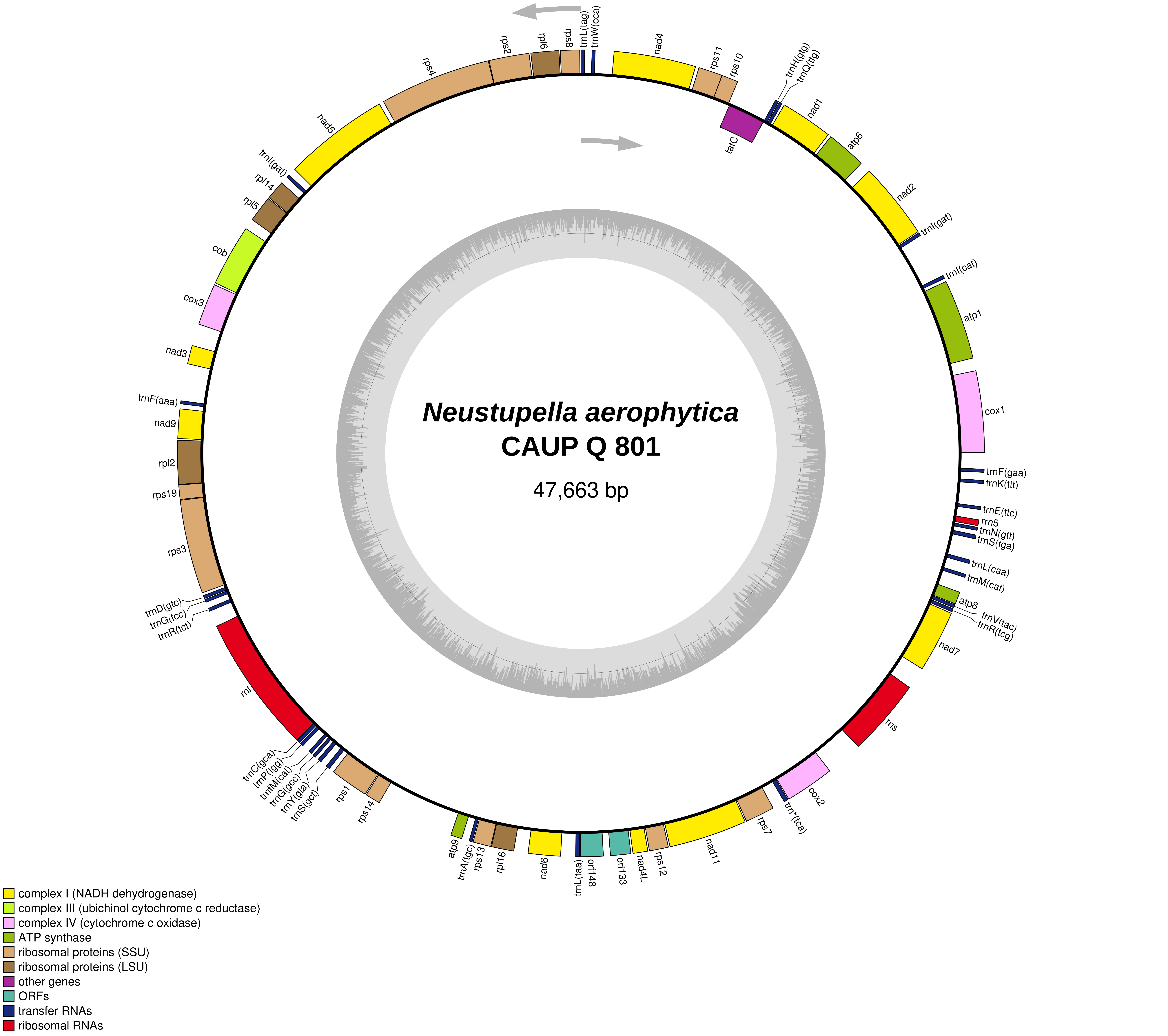

Neustupella aerophytica
CAUP Q 801
47,663 bp
trnL(tag)
trnW(cca)
rps8
rpl6
rps2
rps4
nad5
trnI(gat)
rpl14
rpl5
cob
cox3
nad3
trnF(aaa)
nad9
rpl2
rps19
rps3
trnD(gtc)
trnG(tcc)
trnR(tct)
rnl
trnC(gca)
trnP(tgg)
trnfM(cat)
trnG(gcc)
trnY(gta)
trnS(gct)
rps1
rps14
atp9
trnA(tgc)
rps13
rpl16
nad6
trnL(taa)
orf148
orf133
nad4L
rps12
nad11
rps7
trn*(tca)
cox2
rns
nad7
trnR(tcg)
trnV(tac)
atp8
trnM(cat)
trnL(caa)
trnS(tga)
trnN(gtt)
rrn5
trnE(ttc)
trnK(ttt)
trnF(gaa)
cox1
atp1
trnI(cat)
trnI(gat)
nad2
atp6
nad1
trnQ(ttg)
trnH(gtg)
tatC
rps10
rps11
nad4
complex I (NADH dehydrogenase)
complex III (ubichinol cytochrome c reductase)
complex IV (cytochrome c oxidase)
ATP synthase
ribosomal proteins (SSU)
ribosomal proteins (LSU)
other genes
ORFs
transfer RNAs
ribosomal RNAs

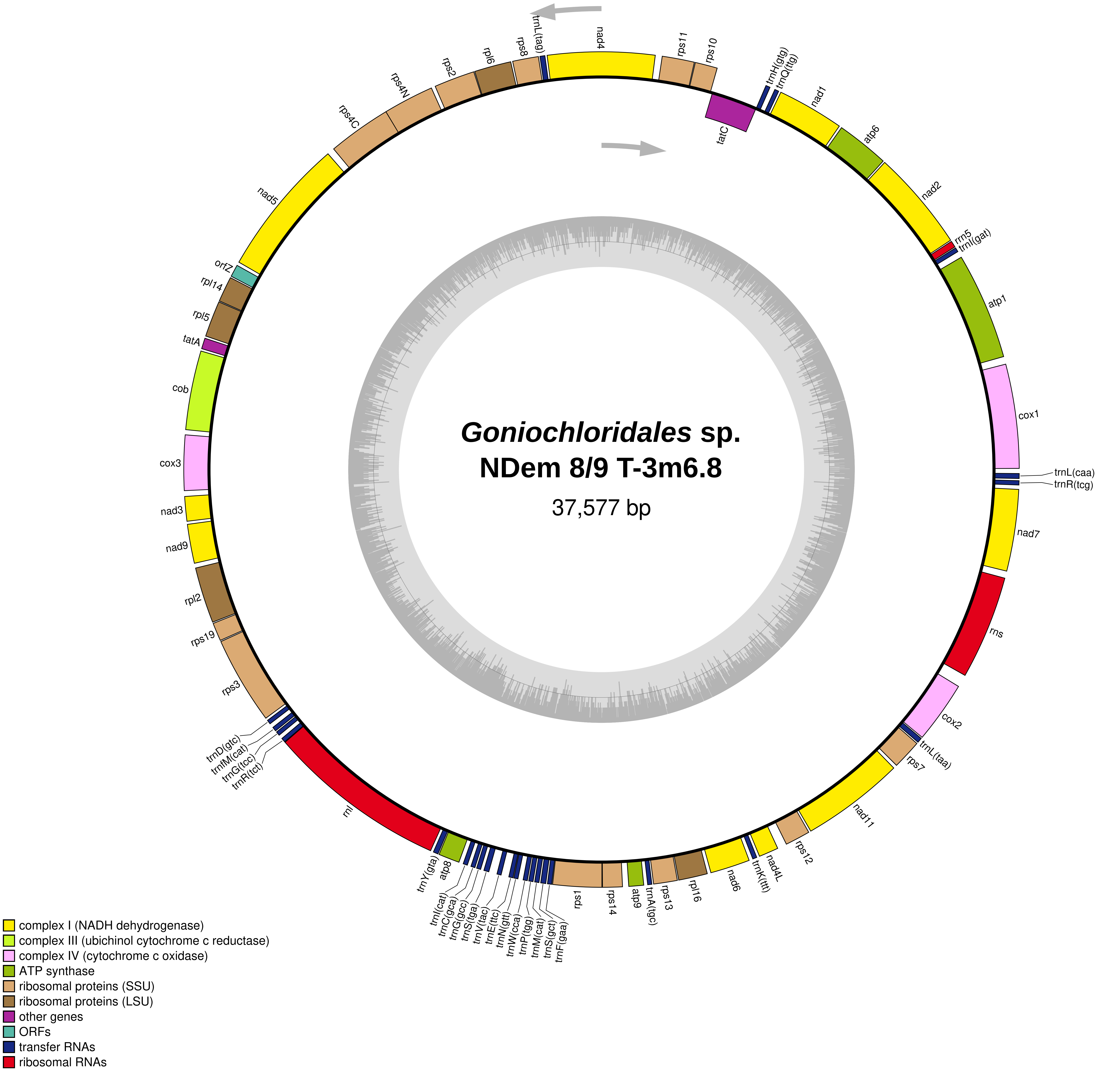

Goniochloridales sp.
NDem 8/9 T-3m6.8
37,577 bp
nad4
rps11
rps10
trnH(gtg)
trnQ(ttg)
tatC
nad1
atp6
nad2
rrn5
trnI(gat)
atp1
cox1
trnL(caa)
trnR(tcg)
nad7
rns
cox2
trnL(taa)
rps7
nad11
rps12
nad4L
trnK(ttt)
nad6
rpl16
rps13
trnA(tgc)
atp9
rps14
rps1
trnF(gaa)
trnS(gct)
trnM(cat)
trnP(tgg)
trnW(cca)
trnN(gtt)
trnE(ttc)
trnV(tac)
trnS(tga)
trnG(gcc)
trnC(gca)
trnI(cat)
atp8
trnY(gta)
rnl
trnR(tct)
trnG(tcc)
trnfM(cat)
trnD(gtc)
rps3
rps19
rpl2
nad9
nad3
cox3
cob
tatA
rpl5
rpl14
orfZ
nad5
rps4C
rps4N
rps2
rpl6
rps8
trnL(tag)
complex I (NADH dehydrogenase)
complex III (ubichinol cytochrome c reductase)
complex IV (cytochrome c oxidase)
ATP synthase
ribosomal proteins (SSU)
ribosomal proteins (LSU)
other genes
ORFs
transfer RNAs
ribosomal RNAs

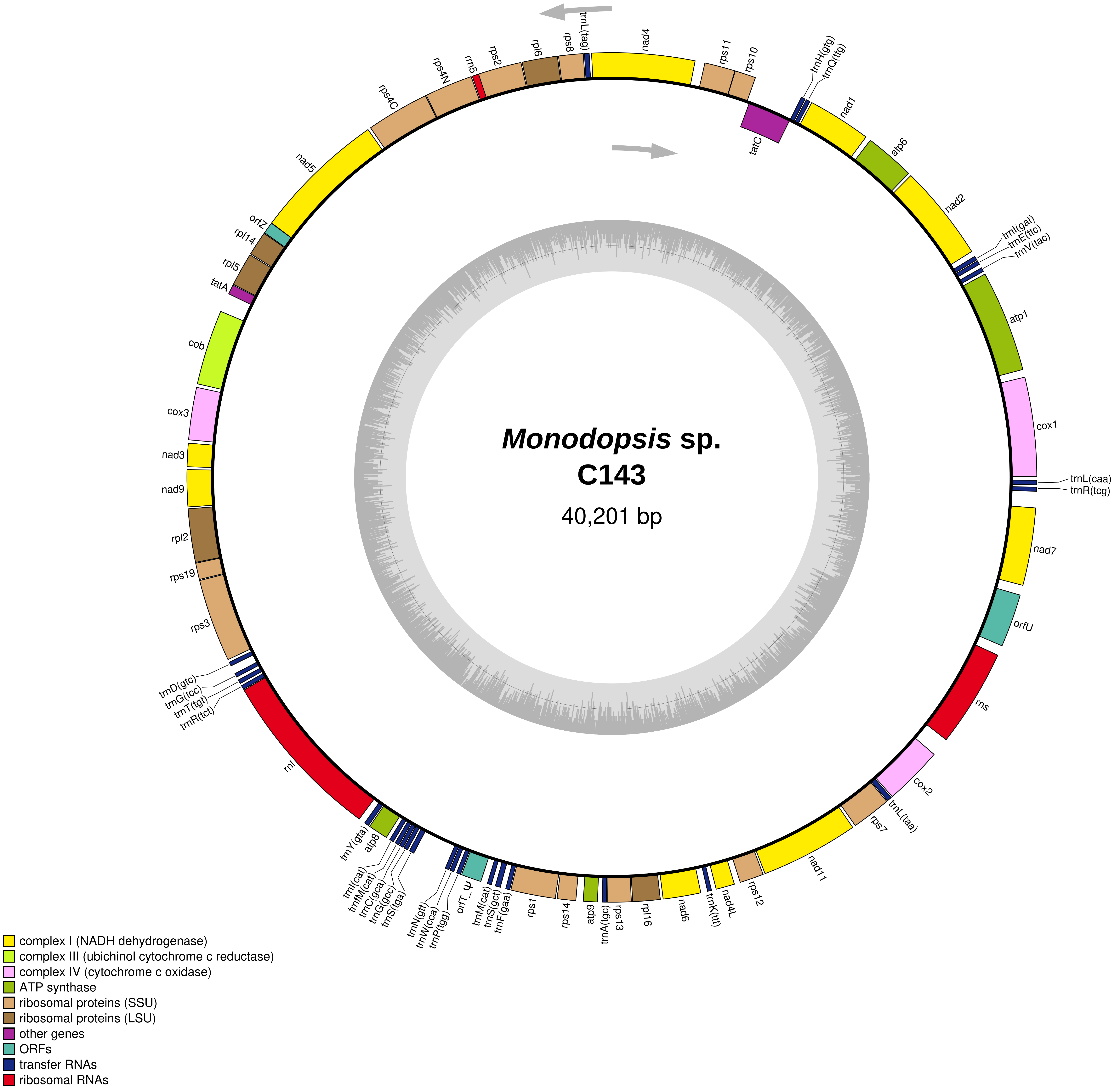

Monodopsis sp.
C143
40,201 bp
trnL(tag)
nad4
rps11
rps10
trnH(gtg)
trnQ(ttg)
tatC
nad1
atp6
nad2
trnI(gat)
trnE(ttc)
trnV(tac)
atp1
cox1
trnL(caa)
trnR(tcg)
nad7
orfU
rns
cox2
trnL(taa)
rps7
nad11
rps12
nad4L
trnK(ttt)
nad6
rpl16
rps13
trnA(tgc)
atp9
rps14
rps1
trnF(gaa)
trnS(gct)
trnM(cat)
orfT_ψ
trnP(tgg)
trnW(cca)
trnN(gtt)
trnS(tga)
trnG(gcc)
trnC(gca)
trnfM(cat)
trnI(cat)
atp8
trnY(gta)
rnl
trnR(tct)
trnT(tgt)
trnG(tcc)
trnD(gtc)
rps3
rps19
rpl2
nad9
nad3
cox3
cob
tatA
rpl5
rpl14
orfZ
nad5
rps4C
rps4N
rrn5
rps2
rpl6
rps8
complex I (NADH dehydrogenase)
complex III (ubichinol cytochrome c reductase)
complex IV (cytochrome c oxidase)
ATP synthase
ribosomal proteins (SSU)
ribosomal proteins (LSU)
other genes
ORFs
transfer RNAs
ribosomal RNAs

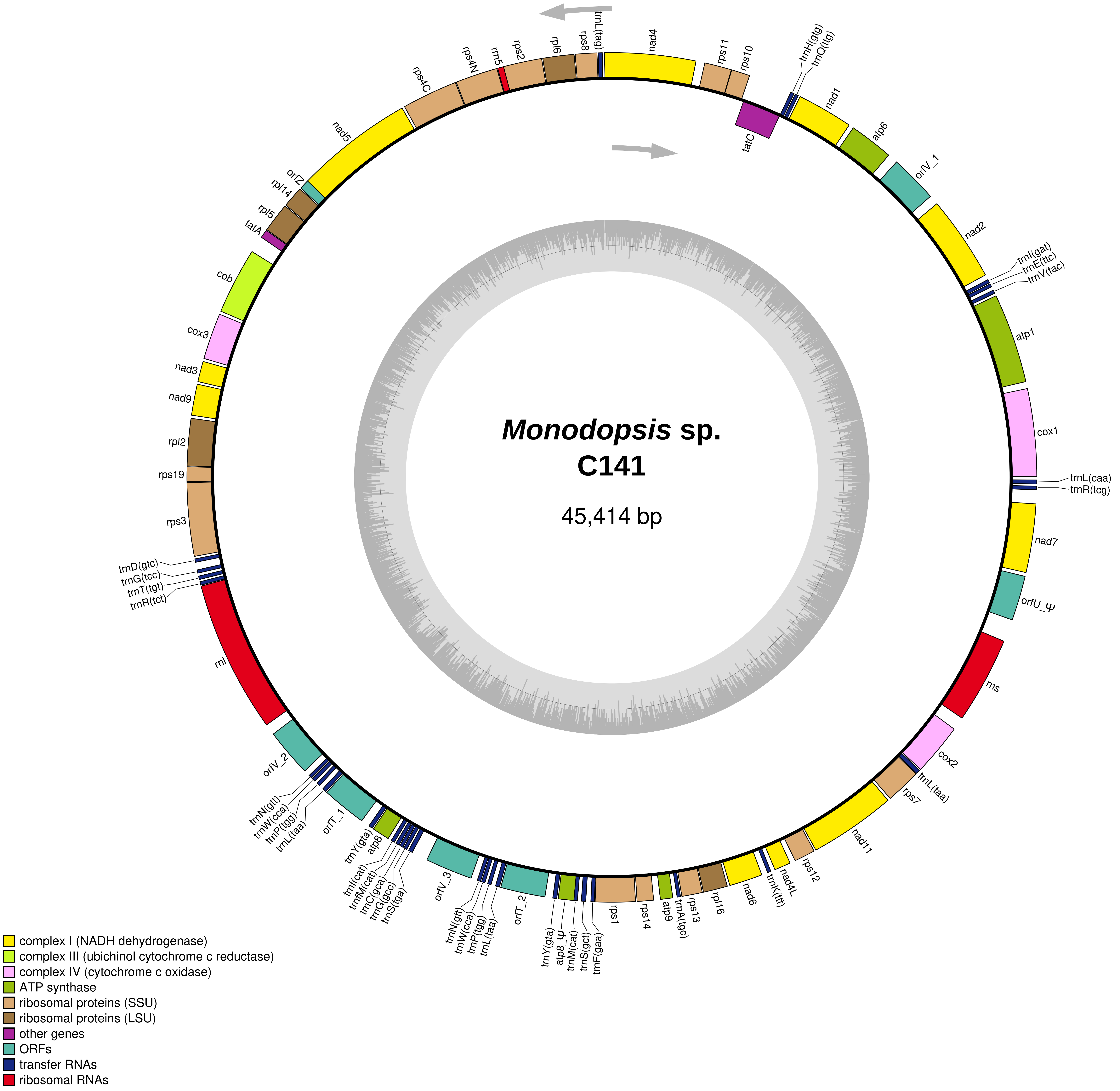

Monodopsis sp.
C141
45,414 bp
trnL(tag)
nad4
rps11
rps10
trnH(gtg)
trnQ(ttg)
tatC
nad1
atp6
orfV_1
nad2
trnI(gat)
trnE(ttc)
trnV(tac)
atp1
cox1
trnL(caa)
trnR(tcg)
nad7
orfU_ψ
rns
cox2
trnL(taa)
rps7
nad11
rps12
nad4L
trnK(ttt)
nad6
rpl16
rps13
trnA(tgc)
atp9
rps14
rps1
trnF(gaa)
trnS(gct)
trnM(cat)
atp8_ψ
trnY(gta)
orfT_2
trnL(taa)
trnP(tgg)
trnW(cca)
trnN(gtt)
orfV_3
trnS(tga)
trnG(gcc)
trnC(gca)
trnfM(cat)
trnI(cat)
atp8
trnY(gta)
orfT_1
trnL(taa)
trnP(tgg)
trnW(cca)
trnN(gtt)
orfV_2
rnl
trnR(tct)
trnT(tgt)
trnG(tcc)
trnD(gtc)
rps3
rps19
rpl2
nad9
nad3
cox3
cob
tatA
rpl5
rpl14
orfZ
nad5
rps4C
rps4N
rrn5
rps2
rpl6
rps8
complex I (NADH dehydrogenase)
complex III (ubichinol cytochrome c reductase)
complex IV (cytochrome c oxidase)
ATP synthase
ribosomal proteins (SSU)
ribosomal proteins (LSU)
other genes
ORFs
transfer RNAs
ribosomal RNAs

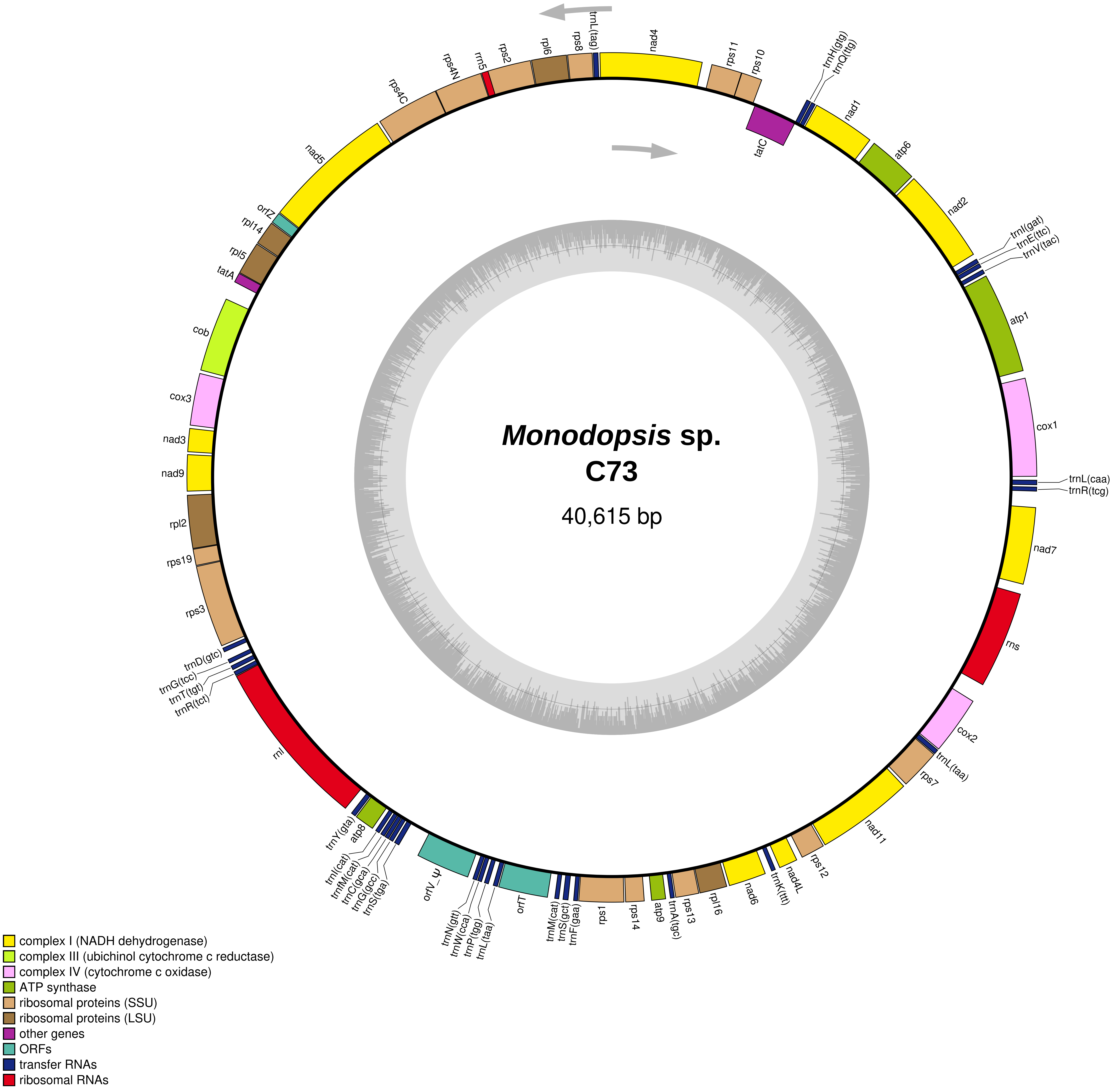

Monodopsis sp.
C73
40,615 bp
trnL(tag)
nad4
rps11
rps10
trnH(gtg)
trnQ(ttg)
nad1
tatC
atp6
nad2
trnI(gat)
trnE(ttc)
trnV(tac)
atp1
cox1
trnL(caa)
trnR(tcg)
nad7
rns
cox2
trnL(taa)
rps7
nad11
rps12
nad4L
trnK(ttt)
nad6
rpl16
rps13
trnA(tgc)
atp9
rps14
rps1
trnF(gaa)
trnS(gct)
trnM(cat)
orfT
trnL(taa)
trnP(tgg)
trnW(cca)
trnN(gtt)
orfV_Ψ
trnS(tga)
trnG(gcc)
trnC(gca)
trnfM(cat)
trnI(cat)
atp8
trnY(gta)
rnl
trnR(tct)
trnT(tgt)
trnG(tcc)
trnD(gtc)
rps3
rps19
rpl2
nad9
nad3
cox3
cob
tatA
rpl5
rpl14
orfZ
nad5
rps4C
rps4N
rrn5
rps2
rpl6
rps8
complex I (NADH dehydrogenase)
complex III (ubichinol cytochrome c reductase)
complex IV (cytochrome c oxidase)
ATP synthase
ribosomal proteins (SSU)
ribosomal proteins (LSU)
other genes
ORFs
transfer RNAs
ribosomal RNAs

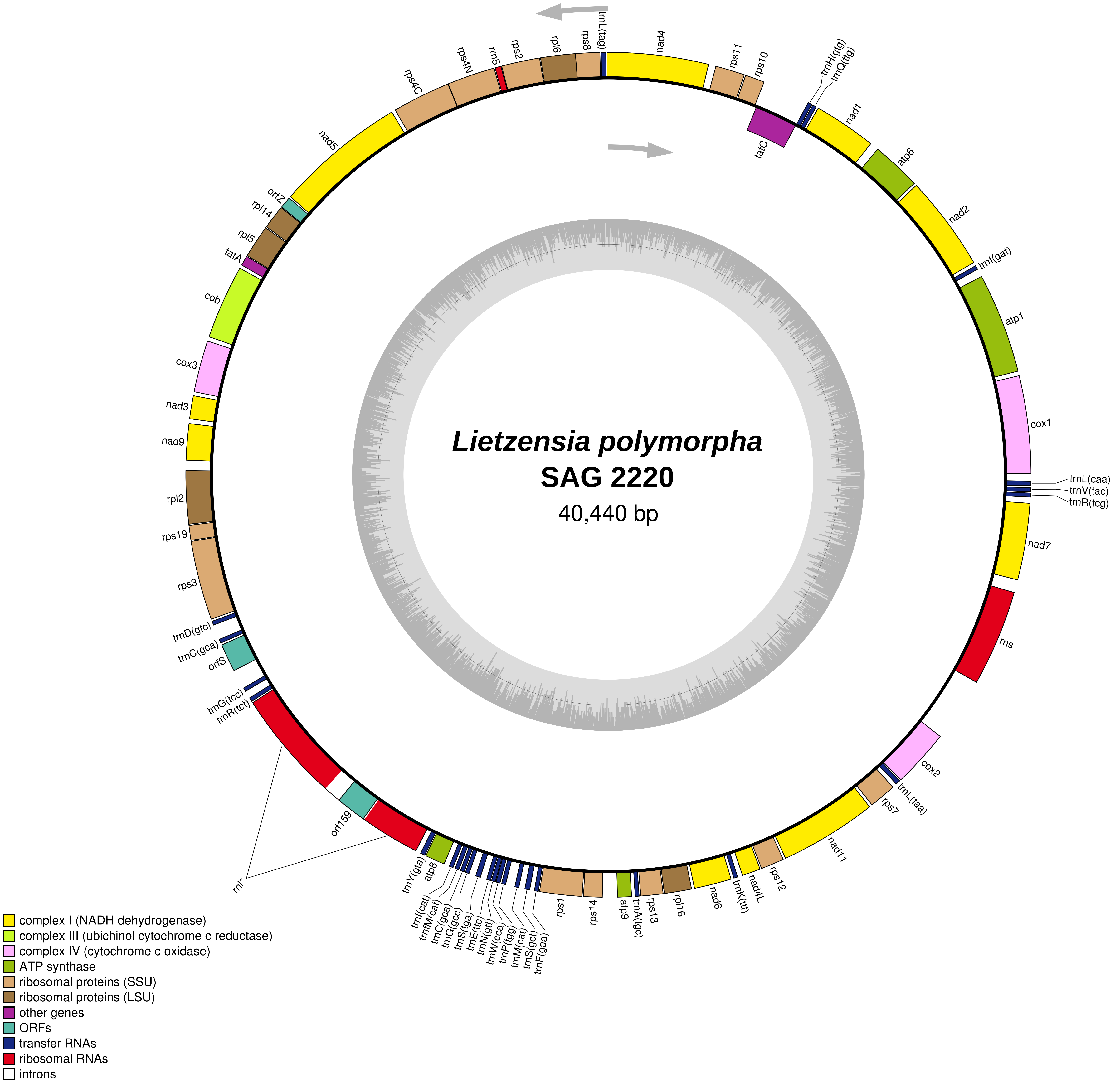
Lietzensia polymorpha
SAG 2220
40,440 bp
trnL(tag)
nad4
rps11
rps10
trnH(gtg)
trnQ(ttg)
tatC
nad1
atp6
nad2
trnI(gat)
atp1
cox1
trnL(caa)
trnV(tac)
trnR(tcg)
nad7
rns
cox2
trnL(taa)
rps7
nad11
rps12
nad4L
trnK(ttt)
nad6
rpl16
rps13
trnA(tgc)
atp9
rps14
rps1
trnF(gaa)
trnS(gct)
trnM(cat)
trnP(tgg)
trnW(cca)
trnN(gtt)
trnE(ttc)
trnS(tga)
trnG(gcc)
trnC(gca)
trnfM(cat)
trnI(cat)
atp8
trnY(gta)
orf159
rnl*
trnR(tct)
trnG(tcc)
orfS
trnC(gca)
trnD(gtc)
rps3
rps19
rpl2
nad9
nad3
cox3
cob
tatA
rpl5
rpl14
orfZ
nad5
rps4C
rps4N
rrn5
rps2
rpl6
rps8
complex I (NADH dehydrogenase)
complex III (ubichinol cytochrome c reductase)
complex IV (cytochrome c oxidase)
ATP synthase
ribosomal proteins (SSU)
ribosomal proteins (LSU)
other genes
ORFs
transfer RNAs
ribosomal RNAs
introns

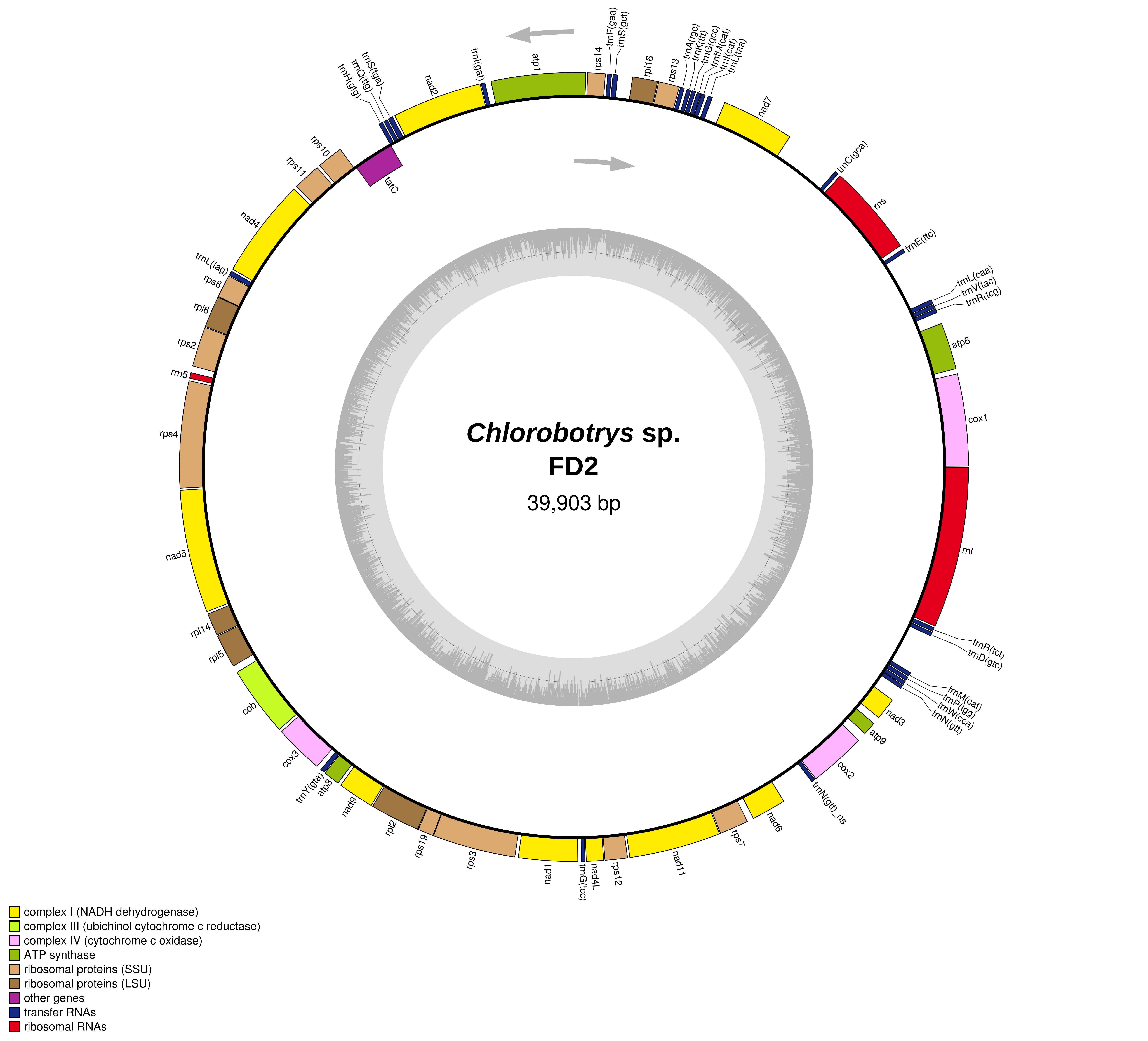

Chlorobotrys sp.
FD2
39,903 bp
atp1
rps14
trnF(gaa)
trnS(gct)
rpl16
rps13
trnA(tgc)
trnK(ttt)
trnG(gcc)
trnfM(cat)
trnI(cat)
trnL(taa)
nad7
trnC(gca)
rns
trnE(ttc)
trnL(caa)
trnV(tac)
trnR(tcg)
atp6
cox1
rnl
trnR(tct)
trnD(gtc)
trnM(cat)
trnP(tgg)
trnW(cca)
trnN(gtt)
nad3
atp9
cox2
trnN(gtt)_ns
nad6
rps7
nad11
rps12
nad4L
trnG(tcc)
nad1
rps3
rps19
rpl2
nad9
atp8
trnY(gta)
cox3
cob
rpl5
rpl14
nad5
rps4
rrn5
rps2
rpl6
rps8
trnL(tag)
nad4
rps11
rps10
tatC
trnH(gtg)
trnQ(ttg)
trnS(tga)
nad2
trnI(gat)
complex I (NADH dehydrogenase)
complex III (ubichinol cytochrome c reductase)
complex IV (cytochrome c oxidase)
ATP synthase
ribosomal proteins (SSU)
ribosomal proteins (LSU)
other genes
transfer RNAs
ribosomal RNAs

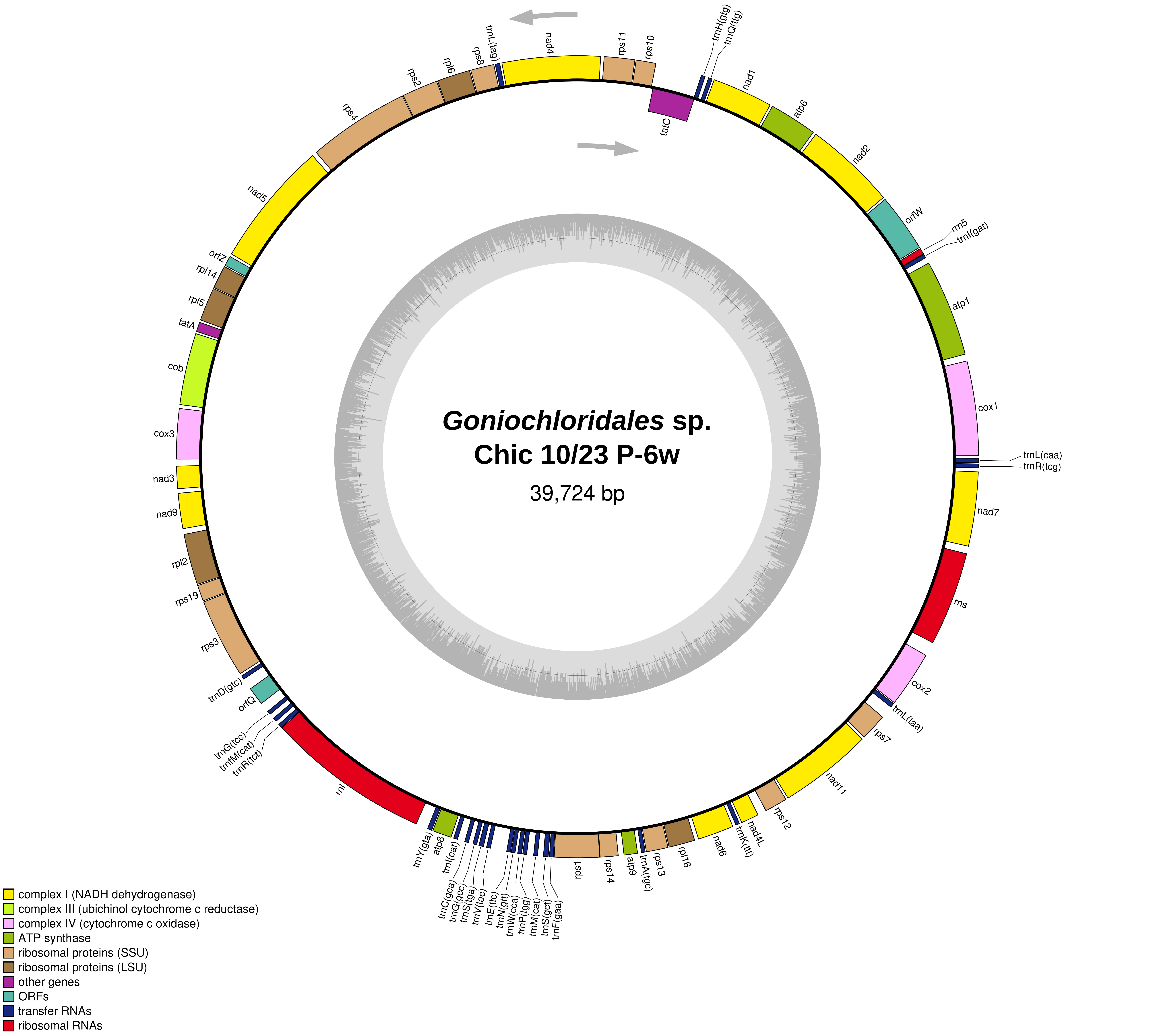
Goniochloridales sp.
Chic 10/23 P-6w
39,724 bp
trnH(gtg)
trnQ(ttg)
nad1
atp6
nad2
orfW
rrn5
trnI(gat)
atp1
cox1
trnL(caa)
trnR(tcg)
nad7
rns
cox2
trnL(taa)
rps7
nad11
rps12
nad4L
trnK(ttt)
nad6
rpl16
rps13
trnA(tgc)
atp9
rps14
rps1
trnF(gaa)
trnS(gct)
trnM(cat)
trnP(tgg)
trnW(cca)
trnN(gtt)
trnE(ttc)
trnV(tac)
trnS(tga)
trnG(gcc)
trnC(gca)
trnI(cat)
atp8
trnY(gta)
rnl
trnR(tct)
trnfM(cat)
trnG(tcc)
orfQ
trnD(gtc)
rps3
rps19
rpl2
nad9
nad3
cox3
cob
tatA
rpl5
rpl14
orfZ
nad5
rps4
rps2
rpl6
rps8
trnL(tag)
nad4
rps11
rps10
tatC
complex I (NADH dehydrogenase)
complex III (ubichinol cytochrome c reductase)
complex IV (cytochrome c oxidase)
ATP synthase
ribosomal proteins (SSU)
ribosomal proteins (LSU)
other genes
ORFs
transfer RNAs
ribosomal RNAs

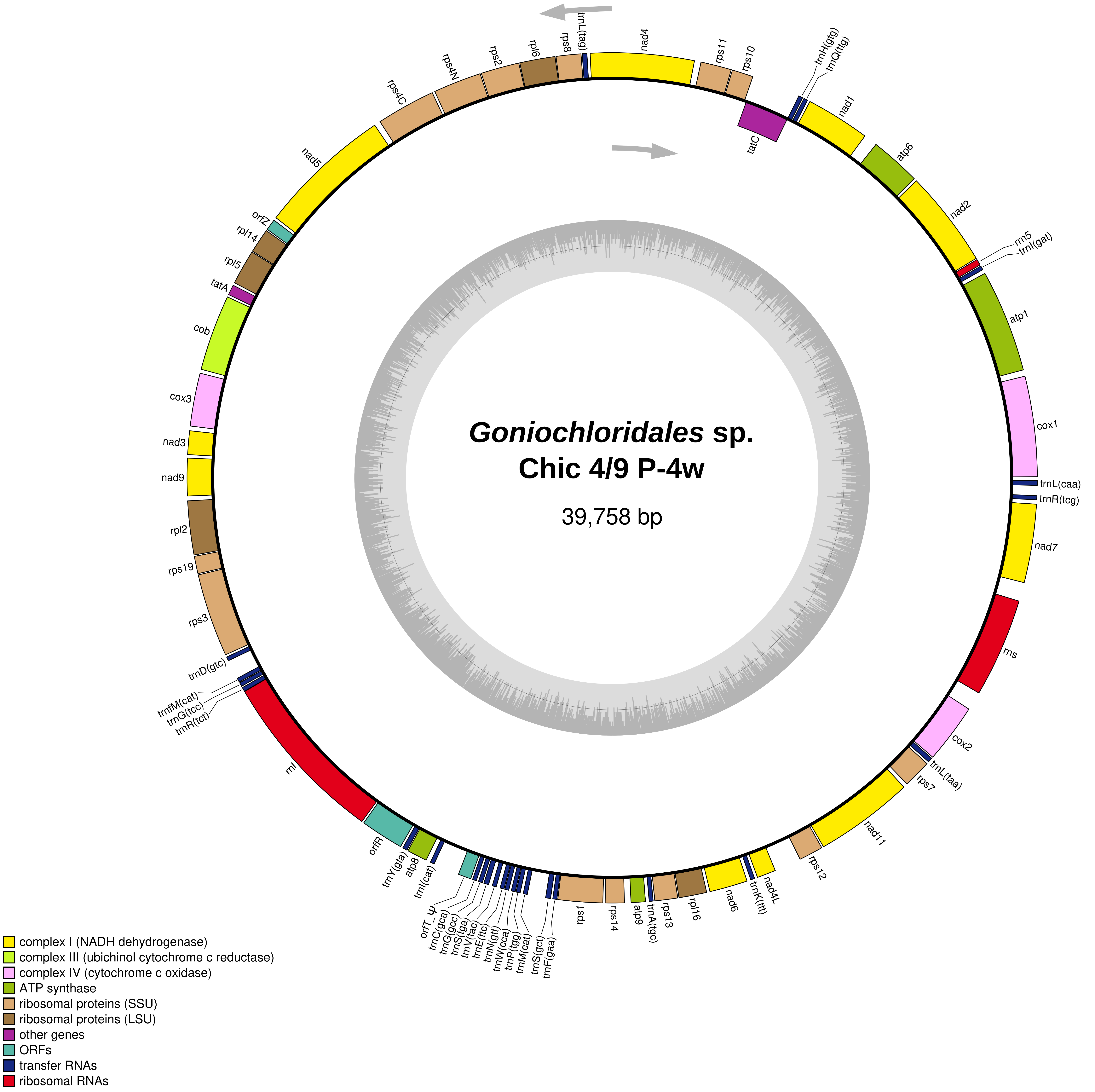

Goniochloridales sp.
Chic 4/9 P-4w
39,758 bp
nad4
rps11
rps10
trnH(gtg)
trnQ(ttg)
tatC
nad1
atp6
nad2
rrn5
trnI(gat)
atp1
cox1
trnL(caa)
trnR(tcg)
nad7
rns
cox2
trnL(taa)
rps7
nad11
rps12
nad4L
trnK(ttt)
nad6
rpl16
rps13
trnA(tgc)
atp9
rps14
rps1
trnF(gaa)
trnS(gct)
trnM(cat)
trnP(tgg)
trnW(cca)
trnN(gtt)
trnE(ttc)
trnV(tac)
trnS(tga)
trnG(gcc)
trnC(gca)
orfT
ψ
trnI(cat)
atp8
trnY(gta)
orfR
rnl
trnfM(cat)
trnG(tcc)
trnR(tct)
trnD(gtc)
rps3
rps19
rpl2
nad9
nad3
cox3
cob
tatA
rpl5
rpl14
orfZ
nad5
rps4C
rps4N
rps2
rpl6
rps8
trnL(tag)
complex I (NADH dehydrogenase)
complex III (ubichinol cytochrome c reductase)
complex IV (cytochrome c oxidase)
ATP synthase
ribosomal proteins (SSU)
ribosomal proteins (LSU)
other genes
ORFs
transfer RNAs
ribosomal RNAs

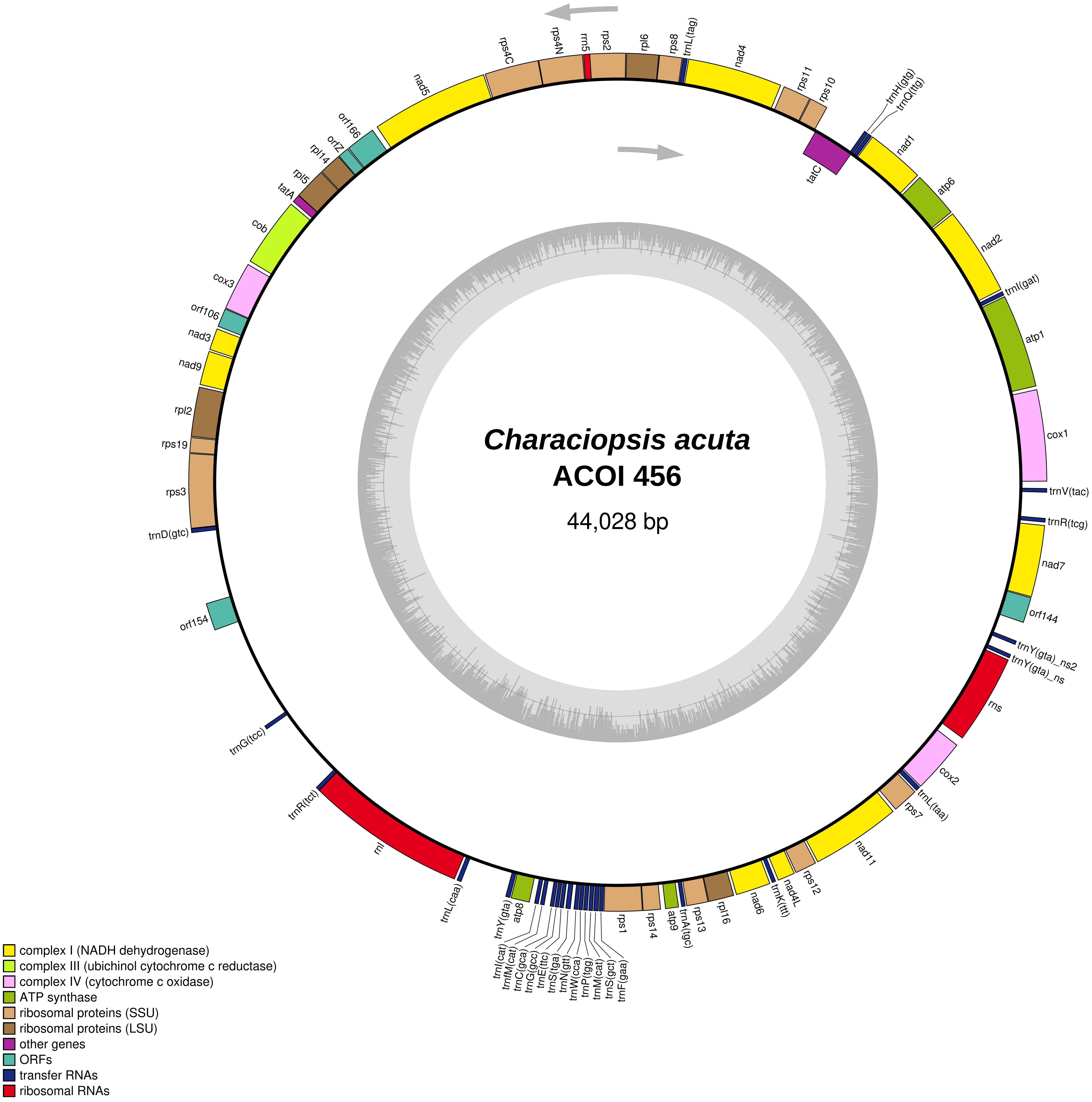

Characiopsis acuta
ACOI 456
44,028 bp
rps4C
rps4N
rrn5
rps2
rpl6
rps8
trnL(tag)
nad4
rps11
rps10
trnH(gtg)
trnQ(ttg)
tatC
nad1
atp6
nad2
trnI(gat)
atp1
cox1
trnV(tac)
trnR(tcg)
nad7
orf144
trnY(gta)_ns2
trnY(gta)_ns
rns
cox2
trnL(taa)
rps7
nad11
rps12
nad4L
trnK(ttt)
nad6
rpl16
rps13
trnA(tgc)
atp9
rps14
rps1
trnF(gaa)
trnS(gct)
trnM(cat)
trnP(tgg)
trnW(cca)
trnN(gtt)
trnS(tga)
trnE(ttc)
trnG(gcc)
trnC(gca)
trnfM(cat)
trnI(cat)
atp8
trnY(gta)
trnL(caa)
rnl
trnR(tct)
trnG(tcc)
orf154
trnD(gtc)
rps3
rps19
rpl2
nad9
nad3
orf106
cox3
cob
tatA
rpl5
rpl14
orfZ
orf166
nad5
complex I (NADH dehydrogenase)
complex III (ubichinol cytochrome c reductase)
complex IV (cytochrome c oxidase)
ATP synthase
ribosomal proteins (SSU)
ribosomal proteins (LSU)
other genes
ORFs
transfer RNAs
ribosomal RNAs

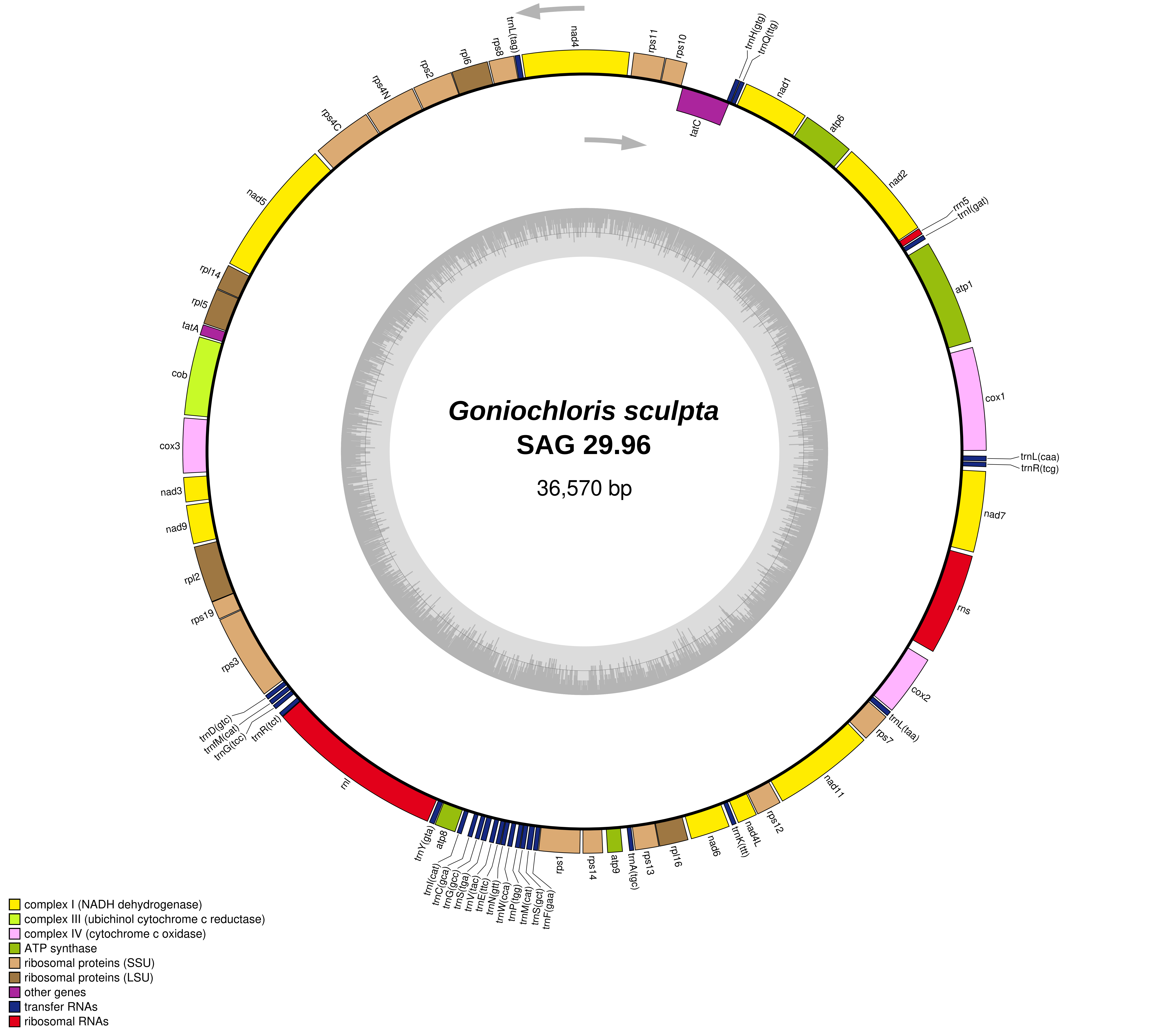
Goniochloris sculpta
SAG 29.96
36,570 bp
nad4
rps11
rps10
trnL(tag)
rps8
rpl6
rps2
rps4N
rps4C
nad5
rpl14
rpl5
tatA
cob
cox3
nad3
nad9
rpl2
rps19
rps3
trnD(gtc)
trnfM(cat)
trnG(tcc)
trnR(tct)
rnl
trnY(gta)
atp8
trnI(cat)
trnC(gca)
trnG(gcc)
trnS(tga)
trnV(tac)
trnE(ttc)
trnN(gtt)
trnW(cca)
trnP(tgg)
trnM(cat)
trnS(gct)
trnF(gaa)
rps1
rps14
atp9
trnA(tgc)
rps13
rpl16
nad6
trnK(ttt)
nad4L
rps12
nad11
rps7
trnL(taa)
cox2
rns
nad7
trnL(caa)
trnR(tcg)
cox1
atp1
rrn5
trnI(gat)
nad2
atp6
nad1
trnH(gtg)
trnQ(ttg)
tatC
complex I (NADH dehydrogenase)
complex III (ubichinol cytochrome c reductase)
complex IV (cytochrome c oxidase)
ATP synthase
ribosomal proteins (SSU)
ribosomal proteins (LSU)
other genes
transfer RNAs
ribosomal RNAs

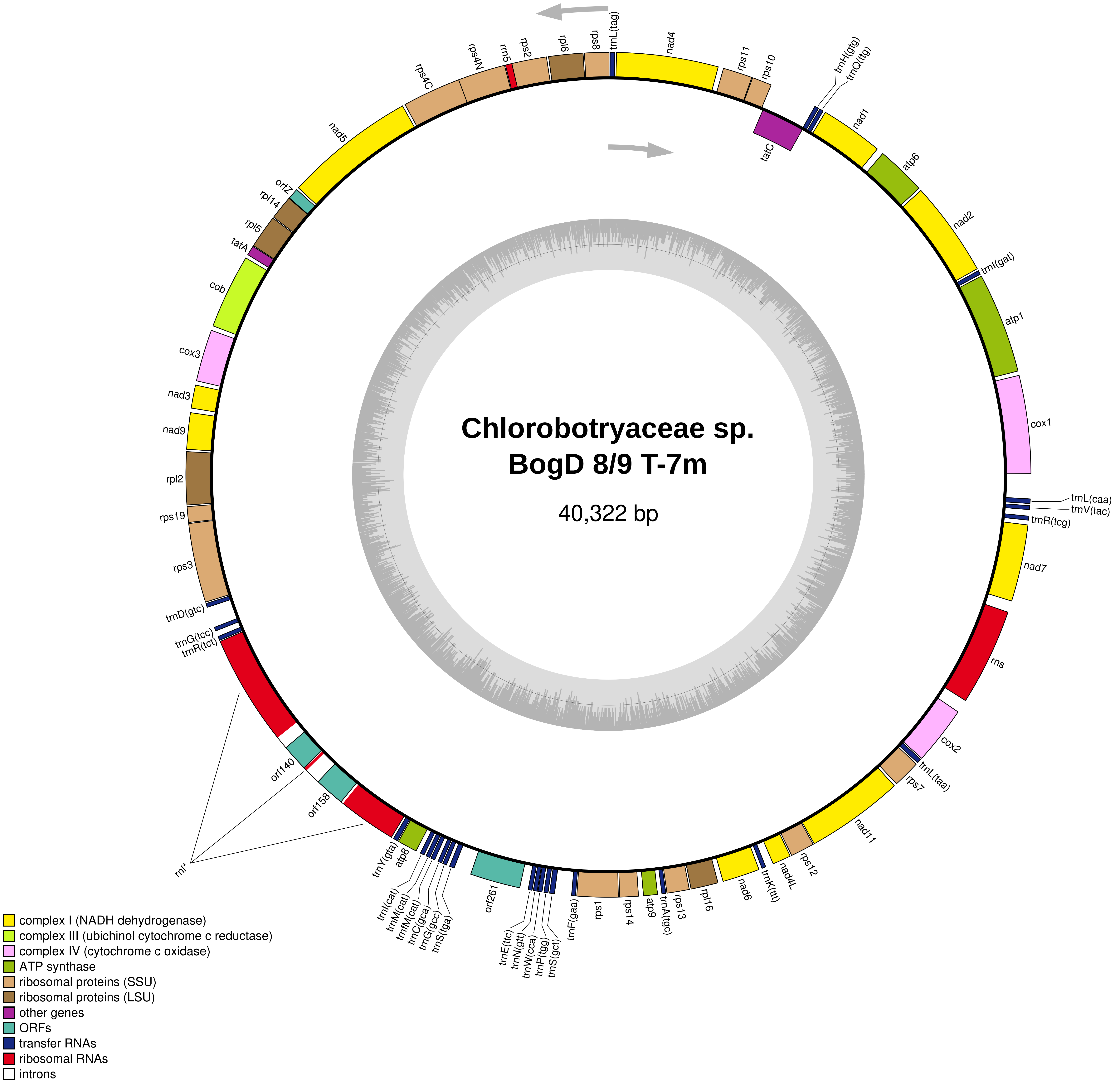

Chlorobotryaceae sp.
BogD 8/9 T-7m
40,322 bp
trnL(tag)
nad4
rps11
rps10
trnH(gtg)
trnQ(ttg)
tatC
nad1
atp6
nad2
trnI(gat)
atp1
cox1
trnL(caa)
trnV(tac)
trnR(tcg)
nad7
rns
cox2
trnL(taa)
rps7
nad11
rps12
nad4L
trnK(ttt)
nad6
rpl16
rps13
trnA(tgc)
atp9
rps14
rps1
trnF(gaa)
trnS(gct)
trnP(tgg)
trnW(cca)
trnN(gtt)
trnE(ttc)
orf261
trnS(tga)
trnG(gcc)
trnC(gca)
trnfM(cat)
trnM(cat)
trnI(cat)
atp8
trnY(gta)
rnl*
orf158
orf140
trnR(tct)
trnG(tcc)
trnD(gtc)
rps3
rps19
rpl2
nad9
nad3
cox3
cob
tatA
rpl5
rpl14
orfZ
nad5
rps4C
rps4N
rrn5
rps2
rpl6
rps8
complex I (NADH dehydrogenase)
complex III (ubichinol cytochrome c reductase)
complex IV (cytochrome c oxidase)
ATP synthase
ribosomal proteins (SSU)
ribosomal proteins (LSU)
other genes
ORFs
transfer RNAs
ribosomal RNAs
introns

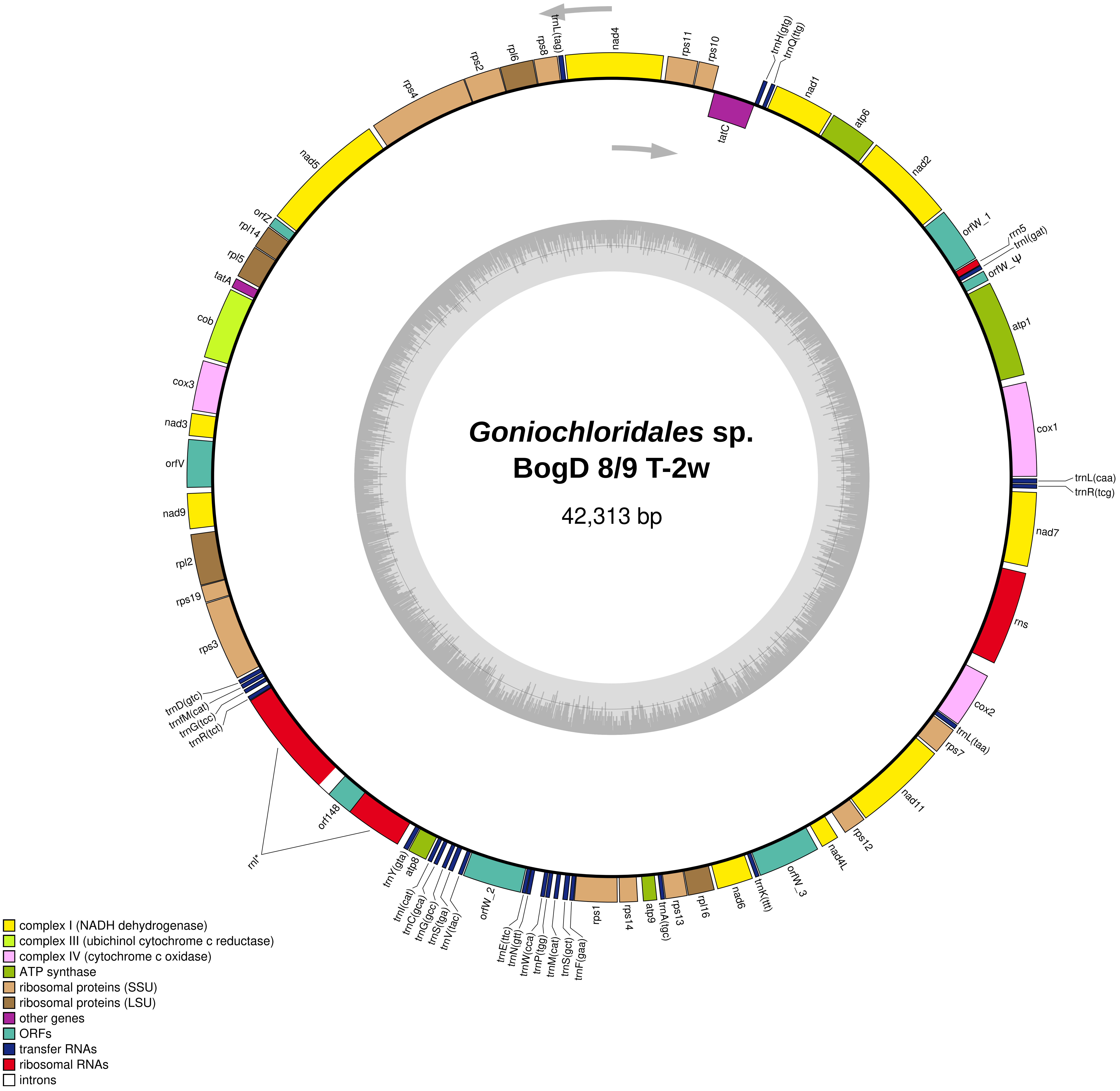
Goniochloridales sp.
BogD 8/9 T-2w
42,313 bp
nad4
rps11
rps10
tatC
trnH(gtg)
trnQ(ttg)
nad1
atp6
nad2
orfW_1
rrn5
trnI(gat)
orfW_Ψ
atp1
cox1
trnL(caa)
trnR(tcg)
nad7
rns
cox2
trnL(taa)
rps7
nad11
rps12
nad4L
orfW_3
trnK(ttt)
nad6
rpl16
rps13
trnA(tgc)
atp9
rps14
rps1
trnF(gaa)
trnS(gct)
trnM(cat)
trnP(tgg)
trnW(cca)
trnN(gtt)
trnE(ttc)
orfW_2
trnV(tac)
trnS(tga)
trnG(gcc)
trnC(gca)
trnI(cat)
atp8
trnY(gta)
rnl*
orf148
trnR(tct)
trnG(tcc)
trnfM(cat)
trnD(gtc)
rps3
rps19
rpl2
nad9
orfV
nad3
cox3
cob
tatA
rpl5
rpl14
orfZ
nad5
rps4
rps2
rpl6
rps8
trnL(tag)
complex I (NADH dehydrogenase)
complex III (ubichinol cytochrome c reductase)
complex IV (cytochrome c oxidase)
ATP synthase
ribosomal proteins (SSU)
ribosomal proteins (LSU)
other genes
ORFs
transfer RNAs
ribosomal RNAs
introns

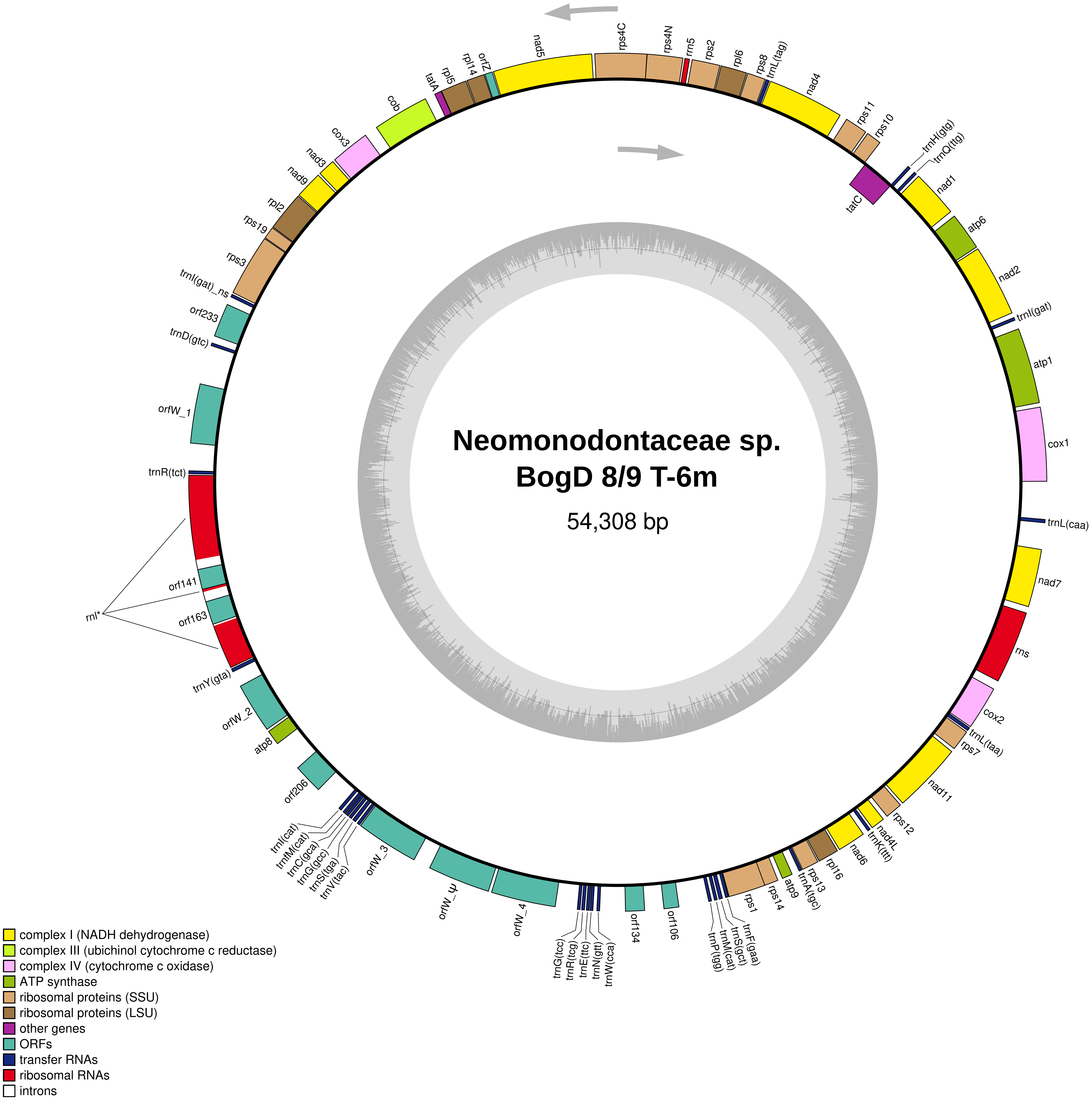

Neomonodontaceae sp.
BogD 8/9 T-6m
54,308 bp
nad5
rps4C
rps4N
rrn5
rps2
rpl6
rps8
trnL(tag)
nad4
rps11
rps10
trnH(gtg)
trnQ(ttg)
tatC
nad1
atp6
nad2
trnI(gat)
atp1
cox1
trnL(caa)
nad7
rns
cox2
trnL(taa)
rps7
nad11
rps12
nad4L
trnK(ttt)
nad6
rpl16
rps13
trnA(tgc)
atp9
rps14
rps1
trnF(gaa)
trnS(gct)
trnM(cat)
trnP(tgg)
orf106
orf134
trnW(cca)
trnN(gtt)
trnE(ttc)
trnR(tcg)
trnG(tcc)
orfW_4
orfW_ψ
orfW_3
trnV(tac)
trnS(tga)
trnG(gcc)
trnC(gca)
trnfM(cat)
trnI(cat)
orf206
atp8
orfW_2
trnY(gta)
orf163
orf141
rnl*
trnR(tct)
orfW_1
trnD(gtc)
orf233
trnI(gat)_ns
rps3
rps19
rpl2
nad9
nad3
cox3
cob
tatA
rpl5
rpl14
orfZ
complex I (NADH dehydrogenase)
complex III (ubichinol cytochrome c reductase)
complex IV (cytochrome c oxidase)
ATP synthase
ribosomal proteins (SSU)
ribosomal proteins (LSU)
other genes
ORFs
transfer RNAs
ribosomal RNAs
introns

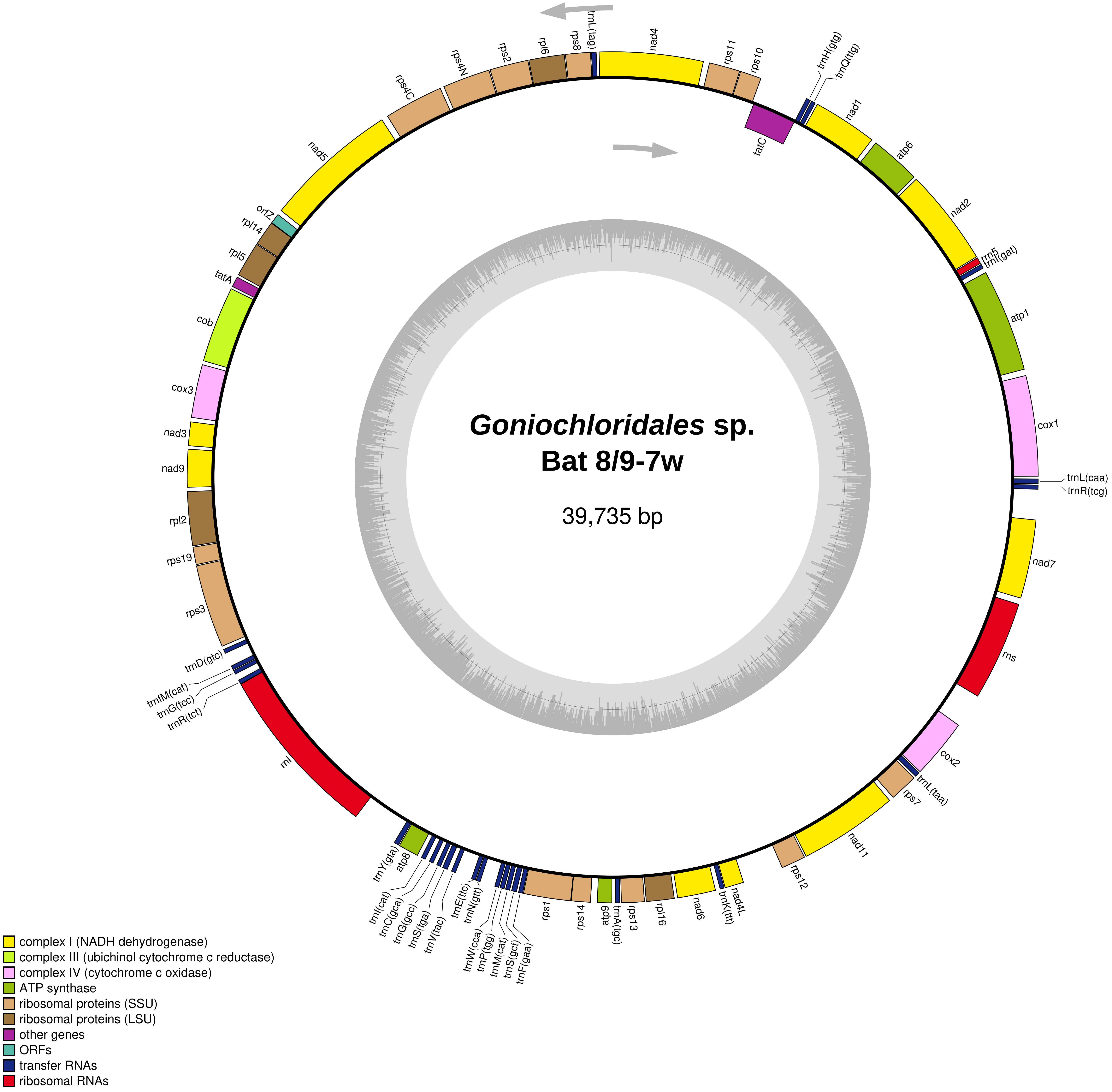

Goniochloridales sp.
Bat 8/9-7w
39,735 bp
nad4
rps11
rps10
trnH(gtg)
trnQ(ttg)
tatC
nad1
atp6
nad2
rrn5
trnI(gat)
atp1
cox1
trnL(caa)
trnR(tcg)
nad7
rns
cox2
trnL(taa)
rps7
nad11
rps12
nad4L
trnK(ttt)
nad6
rpl16
rps13
trnA(tgc)
atp9
rps14
rps1
trnF(gaa)
trnS(gct)
trnM(cat)
trnP(tgg)
trnW(cca)
trnN(gtt)
trnE(ttc)
trnV(tac)
trnS(tga)
trnG(gcc)
trnC(gca)
trnI(cat)
atp8
trnY(gta)
rnl
trnR(tct)
trnG(tcc)
trnfM(cat)
trnD(gtc)
rps3
rps19
rpl2
nad9
nad3
cox3
cob
tatA
rpl5
rpl14
orfZ
nad5
rps4C
rps4N
rps2
rpl6
rps8
trnL(tag)
complex I (NADH dehydrogenase)
complex III (ubichinol cytochrome c reductase)
complex IV (cytochrome c oxidase)
ATP synthase
ribosomal proteins (SSU)
ribosomal proteins (LSU)
other genes
ORFs
transfer RNAs
ribosomal RNAs
